# A proof of concept for continuous, non-invasive, free-living vital signs monitoring to predict readmission following an acute exacerbation of COPD: a prospective cohort study

**DOI:** 10.1186/s12931-022-02018-5

**Published:** 2022-04-26

**Authors:** Grace Hawthorne, Matthew Richardson, Neil J. Greening, Dale Esliger, Samuel Briggs-Price, Emma J. Chaplin, Lisa Clinch, Michael C. Steiner, Sally J. Singh, Mark W. Orme

**Affiliations:** 1 Centre for Exercise and Rehabilitation Science, NIHR Leicester Biomedical Research Centre-Respiratory, University Hospitals of Leicester NHS Trust, Glenfield Hospital, Groby Road, Leicester, LE3 9QP UK; 2 Department of Respiratory Sciences, University of Leicester, Leicester, UK; 3 School of Sport, Exercise and Health Sciences, Loughborough University, Loughborough, UK

**Keywords:** Vital signs, Wearable technology, Digital health, Chronic obstructive pulmonary disease, Physical activity, Skin temperature

## Abstract

**Background:**

The use of vital signs monitoring in the early recognition of an acute exacerbation of chronic obstructive pulmonary disease (AECOPD) post-hospital discharge is limited. This study investigated whether continuous vital signs monitoring could predict an AECOPD and readmission.

**Methods:**

35 people were recruited at discharge following hospitalisation for an AECOPD. Participants were asked to wear an Equivital LifeMonitor during waking hours for 6 weeks and to complete the Exacerbations of Chronic Pulmonary Disease Tool (EXACT), a 14-item symptom diary, daily. The Equivital LifeMonitor recorded respiratory rate (RR), heart rate (HR), skin temperature (ST) and physical activity (PA) every 15-s. An AECOPD was classified as mild (by EXACT score), moderate (prescribed oral steroids/antibiotics) or severe (hospitalisation).

**Results:**

Over the 6-week period, 31 participants provided vital signs and symptom data and 14 participants experienced an exacerbation, of which, 11 had sufficient data to predict an AECOPD. HR and PA were associated with EXACT score (p < 0.001). Three days prior to an exacerbation, RR increased by mean ± SD 2.0 ± 0.2 breaths/min for seven out of 11 exacerbations and HR increased by 8.1 ± 0.7 bpm for nine of these 11 exacerbations.

**Conclusions:**

Increased heart rate and reduced physical activity were associated with worsening symptoms. Even with high-resolution data, the variation in vital signs data remains a challenge for predicting AECOPDs. Respiratory rate and heart rate should be further explored as potential predictors of an impending AECOPD.

*Trial registration:* ISRCTN registry; ISRCTN12855961. Registered 07 November 2018—Retrospectively registered, https://www.isrctn.com/ISRCTN12855961

**Supplementary Information:**

The online version contains supplementary material available at 10.1186/s12931-022-02018-5.

## Introduction

An acute exacerbation of chronic obstructive pulmonary disease (AECOPD) is associated with high risk of premature mortality and a poor long-term prognosis following hospitalisation [1]. AECOPDs can severely impact people’s health-related quality of life [2, 3] and are costly to the healthcare system [4]. The AECOPD readmission rate post-discharge at 30 days and 90 days is 24% and 43% respectively [5].

Pulse oximetry, peak flow and symptoms are commonly used to monitor patients health [6–8]. Respiratory rate, however, has been shown to increase during an exacerbation [9, 10], and has therefore been suggested as a potential target for telemonitoring for the early identification and treatment of AECOPD to prevent hospital admission [10, 11]. Alongside other vital signs, respiratory rate can now be measured passively and continuously but studies measuring respiratory rate have previously been limited to those on long-term oxygen therapy (LTOT) [via built-in O_2_ supplemental devices] [12–14] or devices that require installation or record only once per day [15–17]. In addition to physiological vital signs, behavioural data may provide a signal for health deterioration. Physical activity has been independently associated with increased risk of hospitalisation [18] and readmission [19].

Early management of an AECOPD is associated with a faster recovery [20, 21]. Whilst people living with COPD are able to identify symptomatic changes in their condition, it can be difficult to notice impending severe deterioration [22, 23], and studies assessing patient symptoms continue to show that many AECOPDs are not reported to a clinician despite deterioration in symptoms [24]. Vital signs monitoring has shown some promise in the early recognition of exacerbations [7, 25] but previous research evaluating the use of remote monitoring devices has often been limited by low participant adherence, recall bias and participant burden from multiple devices. Whilst there has been an increase in the use of multiparameter technology to measure vital signs continuously [26], the accuracy of some devices, including pulse oximetry, have been questioned [27] and few have been able to passively, continuously, and non-invasively measure vital signs to predict exacerbations [28].

We aimed to evaluate whether non-invasive, passive, and continuous monitoring of vital signs can (i) identify time-series associations between respiratory rate, heart rate, skin temperature and physical activity and symptoms and (ii) predict an AECOPD and readmission.

## Methods

### Study design

We performed a prospective, observational cohort study of the recovery of people discharged following hospitalisation for an AECOPD. All participants provided written informed consent. This single centre study obtained ethical approval (Research Ethics Committee 15/LO/2055), was registered (ISRCTN12855961), and was undertaken from January 2018–December 2019 at the University Hospitals of Leicester, United Kingdom.

### Recruitment

People admitted to hospital for an AECOPD were recruited when medically fit for discharge. Inclusion criteria were: ≥ 18 years of age, a confirmed clinical diagnosis of COPD from spirometry data in medical records (FEV_1_/FVC < 0.7), and an admission with a primary diagnosis of exacerbation of COPD. Participants were excluded if they had a visual or physical impairment preventing them from wearing the vest, e.g., wheelchair bound or a psychological comorbidity preventing their participation, e.g., dementia, required palliative care or were unable or unwilling to provide written informed consent.

### Measuring free-living vital signs

All participants were asked to wear an Equivital EQ02 + LifeMonitor (Equivital, Cambridge, UK) [29] during waking hours for 6 consecutive weeks (the day after discharge considered day 1). The Equivital LifeMonitor is a vest-like wearable device with a sensor electronic module that sits in a cradle on the side of the LifeMonitor. Participants were asked to remove the LifeMonitor during water-based activities and to charge the device at least every other night. The number of days that the LifeMonitor was worn was examined and wear time was calculated as the average time the LifeMonitor was worn on a single day. The Equivital LifeMonitor measured respiratory rate (RR), heart rate (HR), skin temperature (ST) and physical activity (PA) [30]. RR and HR were calculated as average daily values during wear time. Daily PA was calculated as the proportion of daily wear time when the participant was ambulatory. Further details and the usability and acceptability of the LifeMonitor in a COPD population have previously been reported [31].

### Measuring COPD symptoms

To measure COPD symptoms, participants were asked to complete the Exacerbation of Chronic Pulmonary Disease Tool (EXACT) each day. This 14-item patient-reported questionnaire evaluates breathlessness, cough and sputum and chest symptoms [32, 33], with the total EXACT score ranging from 0 to 100 and higher scores indicating greater symptom severity.

### AECOPD classification

A mild exacerbation was considered a symptom-based AECOPD in accordance with the EXACT scoring; specifically, an increase from baseline of 9 points (for 3 consecutive days) or 12 points (for 2 consecutive days) [32]. A moderate exacerbation was defined by worsening of symptoms where patients required a prescribed course of oral steroids and/or antibiotics. A severe exacerbation was defined by hospitalisation/readmission due to AECOPD.

### Participant characteristics

Demographics, clinical histories, comorbidities, and spirometry data were obtained from medical records or information provided by participants. Height and weight were provided by the participant, obtained from medical records, or measured using a portable stadiometer and weighing scales.

The modified Medical Research Council (MRC) dyspnoea scale [34] was used to measure breathlessness.

### Data processing and statistical analyses

Participants were divided into two groups according to whether they experienced any classification of an AECOPD during the 6-week study period (AECOPD group) or whether they did not (No AECOPD group). Data were analysed and graphs were generated using R version 4.0.0. Continuous variables distributions were assessed for normality. Data are reported as mean (SD) or median (inter-quartile range) and differences between groups were assessed using two sample unpaired T-test or Mann–Whitney U test. Frequency comparisons between groups were assessed using Fisher’s test, with alpha = 0.05.

To investigate associations, a linear mixed model was fitted for EXACT score. Independent variables were RR, HR, ST, PA and time point, and there were no instances of multicollinearity. A random intercept was included for each participant. The linear mixed model was fitted using lmer from the lme4 package [35].

Individual time series plots were analysed to identify the changes in vital signs prior to an exacerbation. Data were analysed at 3 days, 2 days, and the day before an exacerbation, by calculating the percentage change to the first day of an exacerbation. The average percentage change for all participants and the population mean were used to calculate changes in vital signs.

To capture changes in vital signs around the onset of an exacerbation we compared values during stable symptoms, near to an exacerbation and at the onset of exacerbation. These values were taken from; 3 consecutive days where the participant experienced stable symptoms during the study period (stable); 3 days prior to the onset of exacerbation (near exacerbation); and the day of exacerbation onset (exacerbation). Violin plots were used to visualise the group-level data.

## Results

### Participant characteristics

**Fig. 1:**
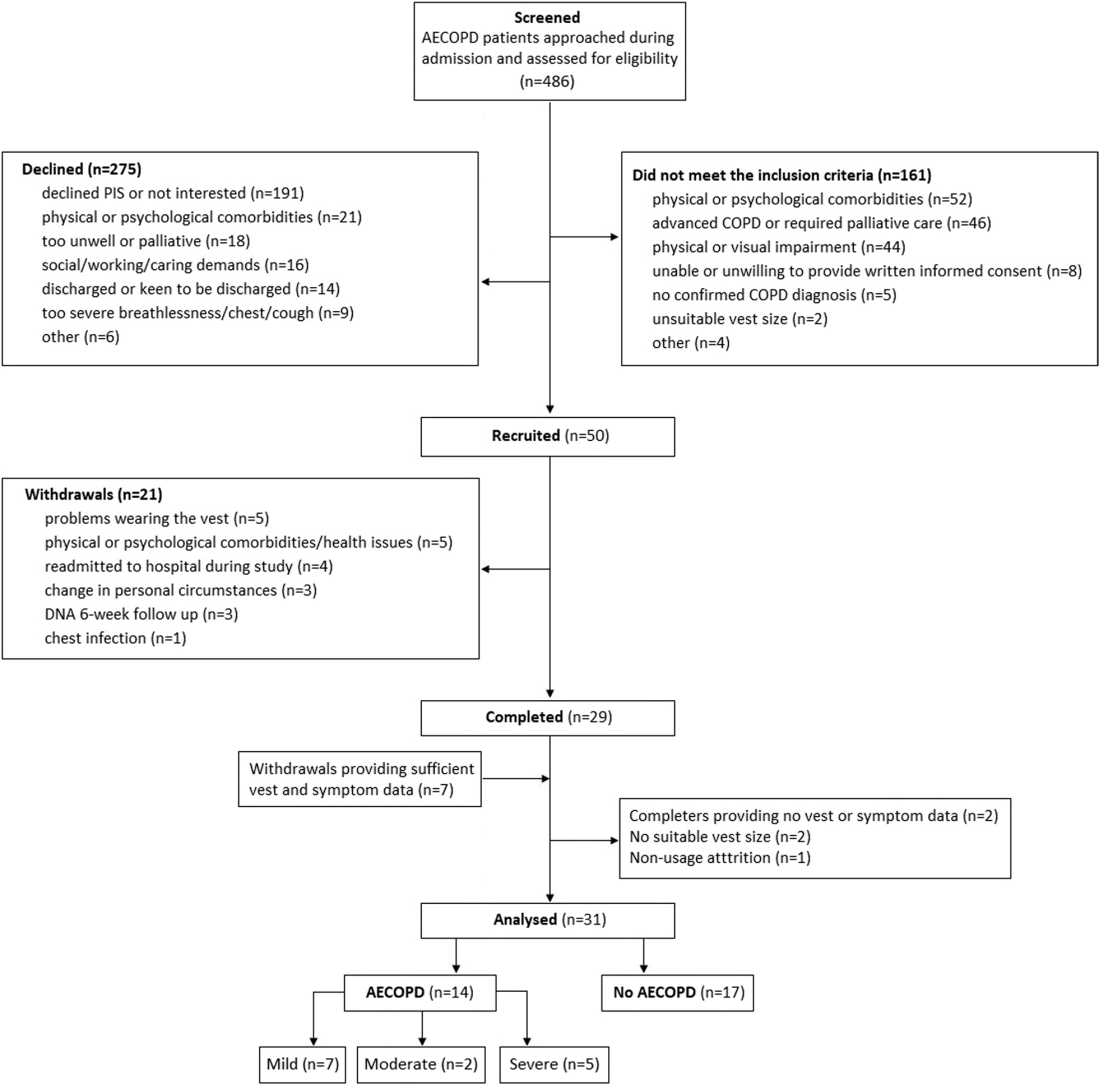
CONSORT flowchart for the study. *AECOPD* acute exacerbation of chronic obstructive pulmonary disease, *DNA* did not attend, *PIS* patient information sheet

Of the 31 participants analysed, 14 participants (45.1%) experienced an AECOPD (Fig. 1). Three participants (21%) experienced > 1 AECOPD during the 6-week study period, so the total number of exacerbations was 18 (10 mild, three moderate and five severe). The No AECOPD group wore the LifeMonitor for significantly fewer days but were otherwise similar to the AECOPD group. In both groups the most prevalent comorbidities were cardiac disease and hypertension, followed by musculoskeletal disorders (Table 1).

**Table 1:** Participant characteristics and vital signs of No AECOPD and AECOPD groups. Data are presented as mean [standard deviation] unless otherwise stated

	No AECOPD (n = 17)	AECOPD (n = 14)
Male, n (%)	8 (52.9)	8 (57.1)
Age (years)	66.5 [8.5]	71.5 [8.1]
BMI (kg/m^2^)	25.9 [7.5]	24.6 [4.4]
FEV_1_ (% predicted)	38.5 [14.7]	48.9 [24.5]
FEV_1_/FVC ratio^a^	0.38 (0.35–0.41)	0.41 (0.31–0.56)
MRC: 2, n (%)	3 (17.6)	3 (21.4)
3, n (%)	3 (17.6)	2 (14.3)
4, n (%)	7 (41.2)	5 (35.7)
5A, n (%)	2 (11.8)	4 (28.6)
5B, n (%)	2 (11.8)	0
Smoking status: never, n (%)	0	0
Ex-smoker, n (%)	12 (70.6)	10 (71.4)
Current, n (%)	5 (29.4)	4 (28.6)
Pack years (years) ^a^	50.0 (45.0–60.0)	42.5 (35.5–63.5)
Home O_2_ use, n (%)	2 (11.8)	2 (14.3)
Hospital admissions in last 12 months^a^	1.0 (0.0–3.0)	2.0 (1.0–2.0)
Exacerbations in last 12 months^a^	1.0 (1.0–1.0)	2.5 (2.0–38.)
Days worn (maximum of 42)^a,^*	28.0 (14.0–37.0)*	38.5 (33.3–41.0)
WT (hrs)	11.7 [1.6]	11.6 [2.3]
RR (breaths/min)	20.9 [3.9]	20.7 [3.5]
HR (bpm)	85.8 [9.3]	82.2 [9.9]
ST (°C)	34.1 [0.8]	34.5 [0.9]
Stationary (hrs/day)	10.2 [1.9]	10.4 [2.0]
PA (% of WT)^a^	10.4 (9.6–17.0)	8.9 (7.9–13.9)
PA (hrs/day)^a,b^	1.2 (0.9–2.1)	1.1 (0.7–1.5)
EXACT score	41.5 [11.9]	42.6 [12.9]
*Comorbidities n (%)*		
Cardiac disease	7 (41.2)	4 (33.3)
Hypertension	8 (47.1)	4 (33.3)
Diabetes	4 (23.5)	0
Kidney disease	1 (5.9)	0
Musculoskeletal disorders	5 (29.4)	2 (29.4)
Mental health disorders	4 (23.5)	0

*BMI* Body Mass Index, *EXACT* Exacerbations of Chronic Pulmonary Disease Tool, *FEV1* forced expiratory volume in 1 s, *FVC* forced vital capacity, *HR* heart rate, *MRC* Medical Research Council, *O*_*2*_ Oxygen, *PA* physical activity, *RR* respiratory rate, *ST* skin temperature, *WT* wear time

^a^Data are presented as median (interquartile range). An unpaired T-test was used for parametric data and Mann–Whitney U test was used for non-parametric data. ^b^Calculated using the Equivital LifeMonitor. *p < 0.05

### Associations between EXACT and vital signs

Results for the mixed model are shown in Table 2, and the full output for the model is shown in Additional file 2: Tables S1. A statistically significant association was found between HR and EXACT score (coefficient, 95% CI, p value; 0.27, 0.16 to 0.33, p < 0.001) and PA was shown to be negatively associated with EXACT score (− 0.22, − 0.33 to − 0.09, p < 0.001) (Table 2).

**Table 2:** Multilevel linear regression model identifying the associations between vital signs and symptom severity

Vital sign	Coefficient	95% CI	P value
*EXACT Score*			
RR	0.25	− 0.13 to 0.59	0.205
HR	0.27	0.16 to 0.33	< 0.001
ST	0.83	− 0.15 to 1.69	0.100
PA	− 0.22	− 0.33 to -0.09	< 0.001

*CI* confidence interval, *EXACT* Exacerbations of Chronic Pulmonary Disease Tool, *HR* heart rate, *PA* physical activity, *RR* respiratory rate, *ST* skin temperature

### Predicting an AECOPD post-discharge

#### Prior to AECOPD

Of the 18 exacerbations, four (22%) occurred too early post-discharge to obtain sufficient data to analyse prior to the exacerbation, and a further three participants (17%) did not provide data in the 3 days prior to the exacerbation, leaving 11 exacerbations that could be analysed during this period. Seven of the 11 exacerbations had increased RR 3 days prior to the onset of exacerbation (mean[SD]; 2.0 [0.2] breaths/min). Nine of 11 exacerbations showed an increase in HR from 3 days prior to the onset of exacerbation (8.1 [0.7] bpm). There were no noticeable changes for ST or PA. Prior to an exacerbation, seven exacerbations showed an increase in ST (0.5 [0.01] °C), four exacerbations showed a decrease in ST (− 1.0 [0.03] °C), five exacerbations showed an increase in PA (0.9 [0.4] hrs) and six exacerbations showed a decrease in PA (− 0.3 [0.1] hrs).

**Fig. 2:**
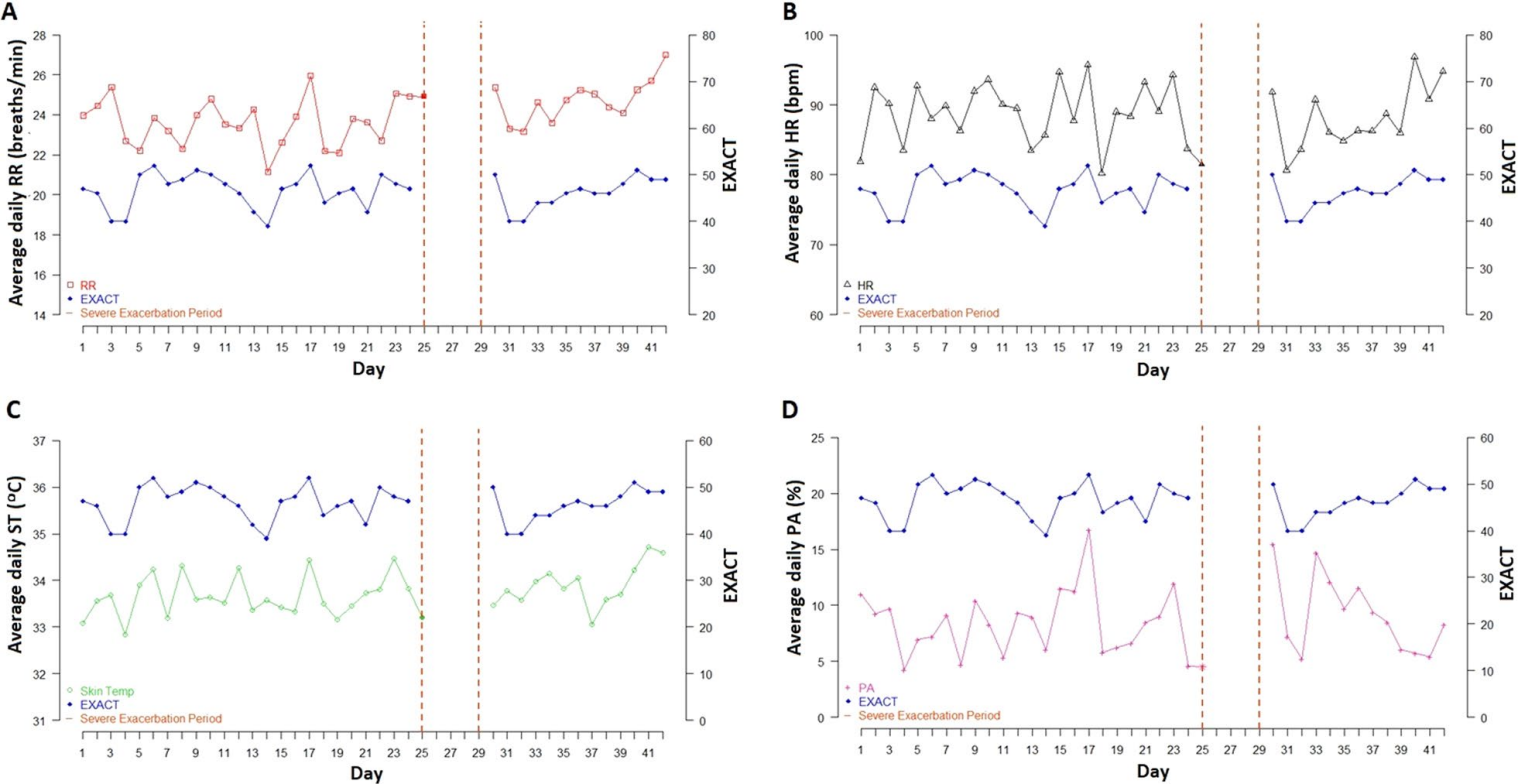
Time series plots for a single participant who experienced a severe exacerbation. **A** RR and EXACT; **B** HR and EXACT; **C** ST and EXACT; **D** PA and EXACT. *EXACT* Exacerbations of Chronic Pulmonary Disease Tool, *HR* heart rate, *PA* physical activity, *RR* respiratory rate, *ST* skin temperature

Figure 2 shows the time series plots for RR, HR, ST and PA against EXACT score for a single participant who experienced a severe exacerbation. All individual plots and the collective summary can be found in Additional file 1: Fig. S2 and Additional file 3: Table S3, respectively.

#### During an AECOPD

Some participants continued to wear the LifeMonitor during the exacerbation period, where seven of the 18 exacerbations (39%) provided sufficient data. All seven exacerbations had an increase in RR during the exacerbation period (mean[SD]; 0.8 [0.1] breaths/min), and HR was seen to increase during the exacerbation period for six of the seven exacerbations (6.7 [0.9] bpm). For ST, four of the seven exacerbations had an increase in ST during the exacerbation period (0.3 [0.01] °C) and three had a decrease in ST (− 1.2 [0.01] °C). Three of the seven exacerbations had an increase in PA during the exacerbation period (0.9 [0.4] hrs) and four had a decrease in PA (− 0.2 [0.1] hrs).

### Group analysis

**Fig. 3:**
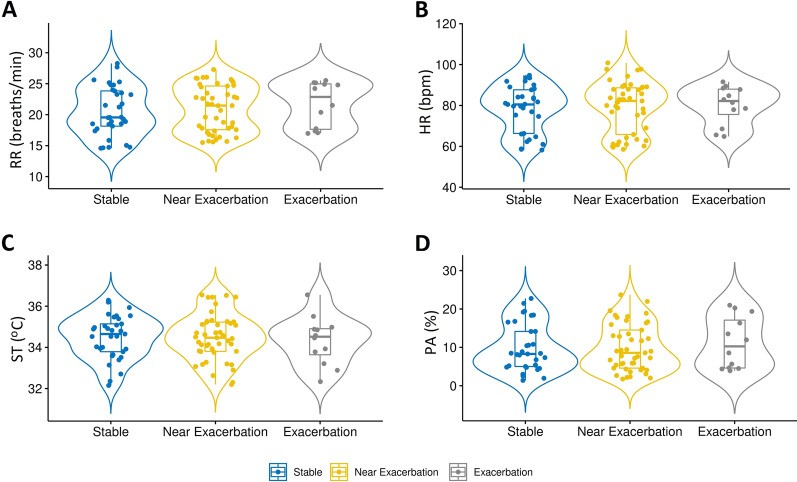
Violin plots showing the distribution of; **A** RR; **B** HR; **C** ST; **D** PA across the three time points. (n = 15). Violin plots are composed of a central box plot surrounded by a border corresponding to an estimate of the probability distribution. The three time points are; 3 days when symptoms where stable (Stable); 3 days prior to an exacerbation (Near Exacerbation); and the onset of an exacerbation (Exacerbation). *HR* heart rate, *PA* physical activity, *RR* respiratory rate, *ST* skin temperature

For the 15 exacerbations that could be analysed, there was very little change in HR, ST, and PA between stable symptoms, near to exacerbation and the onset of exacerbation (Fig. 3). RR increased from stable symptoms to near exacerbation, and further increased to the onset of exacerbation (median[IQR]; 19.6[5.7] to 21.5[7.1] to 22.9[7.3]; Fig. 3). The distribution for RR appears not to be consistent across all participants. There was notable variation for all vital signs across all time points.

## Discussion

Heart rate and physical activity were associated with symptom severity (EXACT score). Exploring individuals’ changes in vital signs prior to an AECOPD showed that in nine out of 11 exacerbations heart rate increased on average by 8 bpm and in 7 out of 11 exacerbations respiratory rate increased on average by 2 breaths/min. The predictive capabilities of statistical models for the group were limited by variation in vital signs between individuals and the limited sample size. Thus, respiratory rate and heart rate should be further explored as potential predictors of future AECOPDs post-discharge.

### Respiratory rate

Results from the present study suggest that respiratory rate was not associated with symptoms over the 6-week study period. However, analysis prior to an AECOPD suggests that respiratory rate may be useful in predicting an AECOPD. Respiratory rate telemonitoring to predict an AECOPD has shown inconsistent results [11–16, 25, 36]. Our findings are in line with other studies that have measured respiratory rate continuously in those receiving LTOT, as Yanez et al. [14] observed a change in respiratory rate from baseline, with a 15–30% increase (2.3–4.4 breaths/min) 24–48 h before hospitalisation. Blouet et al. [12] reported a smaller increase of 2.1 breaths/min across 10 days preceding admission to hospital for a severe AECOPD. In the present study, on average, respiratory rate increased by 2.0 breaths/min 3 days prior to exacerbation and by 2.2 breaths/min the day before the onset. Most previous studies that have remotely monitored respiratory rate have typically recruited people with stable COPD symptoms to predict exacerbations [12–15], and we have observed a similar increase in respiratory rate for those experiencing an AECOPD during recovery from hospitalisation. Vital sign variation between individuals makes their predictive capabilities more challenging [6], however, respiratory rate warrants further exploration as a possible early predictor of readmission post-discharge.

In the present study, all participants who wore the LifeMonitor during the exacerbation period experienced an increase in respiratory rate. Our results are in accordance with other studies that have observed an increase in respiratory rate for patients hospitalised for an AECOPD [10, 11]. Although respiratory rate is the most common objective measurement of respiration, there are other respiratory changes associated with an AECOPD and recovery. For example, lung mechanics [37], respiratory sounds [38] and neural respiratory drive [17] have been assessed in an AECOPD population but difficulties still remain in the use of non-invasive, continuous measurement modalities that are accepted by people with COPD.

### Heart rate

In the present study, HR values over the six weeks were associated with symptoms and changes in HR could possibly predict an AECOPD. Previous studies have provided mixed results regarding the use of HR in predicting exacerbations, with some studies suggesting that HR cannot predict an AECOPD [36, 39]. Our results are in accordance with other studies that identified HR as a useful vital sign for predicting exacerbations [6, 7, 15, 40]. The present study found that HR increased by 8 bpm 3 days prior to exacerbation onset; similar to Burton et al. [6], Rajeh et al. [40] and Hurst et al. [7] (increase of 7 bpm at the start of exacerbation, increase of 7 bpm the day before and 7 bpm 3 days before an AECOPD), and greater than Shah et al. [15] (3 bpm increase in the week prior to exacerbation onset). The assessment of patient symptoms is varied among studies, and as such a more comprehensive symptom diary may provide more detail regarding changes in patients health. The increase in respiratory rate prior to an AECOPD observed in the present study and other studies [6, 7, 15, 40], may be due to the identification of milder exacerbations via a detailed symptom diary.

Both patients and clinicians express considerable confidence in pulse oximetry [22], and as such many studies have remotely monitored both HR and oxygen saturation (SpO_2_) in combination using pulse oximetry [6, 7, 15, 40]. Whilst some studies have highlighted the use of pulse oximetry for predicting AECOPDs [7, 25, 36], measurements are often limited to once daily as practical issues of continuous and non-invasive monitoring exist. Blood pressure (BP) has also been assessed as a vital sign to predict exacerbations and hospital admissions [41], however, similar to SpO_2_, it is impractical to continuously measure BP during free-living conditions. It should be noted that the Equivital LifeMonitor used in the present study can monitor SpO_2_ and BP using additional devices connected to the vest. However, other studies have shown participants prefer devices without a pulse-oximeter probe [42], and at the time of the study additional devices were not deemed feasible according to patient and public involvement feedback and would have had implications of battery and memory life restricting extended monitoring periods. Given the more recent advances in cuffless BP measurements, Hosanee et al. [43] have suggested that a wrist device may be the most appropriate cuffless BP measurement, and although a wrist device is often preferred by people living with COPD [28], there are technical implications for measuring heart rate and respiratory rate [11]. Future integration of wearable technology remains a challenge due to the difficulties in capturing SpO_2_, BP, respiratory rate, heart rate, skin temperature and physical activity measurements with a single, non-invasive device.

### Skin temperature and physical activity

The present study identified no associations between skin temperature and symptoms or experiencing an AECOPD, and no associations between physical activity and experiencing an AECOPD. In line with previous studies examining body temperature [36, 39], we observed no notable changes in skin temperature prior to an AECOPD which may be caused by measurements tending towards the ambient or inner clothing temperature due to the nature of the technology. As a strong predictor of mortality in COPD, physical activity is a core component of self-management interventions [44], and it has now become a behavioural vital sign that is commonly measured in a COPD population [33, 45–47]. Similar to a previous study that assessed physical activity using a pedometer and symptoms via the EXACT diary [33], the present study observed an association between worsening symptoms and a decrease in physical activity. However, there were no notable changes in physical activity leading up to an AECOPD. Physical activity is characteristically low in a COPD population, particularly post-AECOPD [45, 46], suggesting a floor-effect may exist, whereby individuals have already minimised physical activity levels. This emphasises the need for early identification of exacerbations to allow patients to maintain their physical activity levels and overall health-related quality of life.

### Study limitations

A limitation of the study is the sample attrition due to the study being conducted during a challenging period for individuals post-AECOPD, which has been reflected in other research [48–50] and in interventions such as post-exacerbation Pulmonary Rehabilitation [5]. Healthier individuals are more likely to consent to research participation and as such there is potential selection bias which may contribute towards our low readmission rate. From visual inspection of individual participant data, we generated further associations between vital signs and exacerbations, although the small sample size limited our ability to stratify by severity of exacerbation. Measuring vital signs overnight may have provided more consistent ‘baseline’ values for vital signs, helping to reduce observed variation. Across all studies, the lack of consensus in the classifications of AECOPD and baseline values makes it challenging to perform direct comparisons.

## Conclusions

Continuous and non-invasive monitoring of vital signs during the post-discharge period showed that increased heart rate and reduced physical activity were associated with greater symptom severity. At the individual level, respiratory rate and heart rate cannot be ruled out as potential predictors of an impending AECOPD and should be further explored in relation to preventing readmissions and exacerbations. The predictive capabilities of the data are hindered by the large individual variation of vital signs measured in the free-living environment.

## Supplementary Information

**Additional file 1: Figure S2.** Individual plots for respiratory rate, heart rate, skin temperature and physical activity for all participants.

**Additional file 2: Table S1.** Linear Mixed Model for all variables and EXACT score.

**Additional file 3: Table S3.** Collective summary of individual participant data, 3 days-, 2 days- and the day before the onset of exacerbation.

## Data Availability

The data that support the findings of this study are available from the corresponding author upon reasonable request.

## Abbreviations

AECOPD: Acute Exacerbation of Chronic Obstructive Pulmonary Disease
BMI: Body Mass Index
CI: Confidence interval
COPD: Chronic obstructive pulmonary disease
DNA: Did not attend
EXACT: Exacerbations of Chronic Pulmonary Disease Tool
FEV_1_: Forced expiratory volume in 1 s
FVC: Forced vital capacity
HR: Heart rate
IQR: Interquartile range
MRC: Medical Research Council
O_2_: Oxygen
PA: Physical activity
PIS: Patient information sheet
PR: Pulmonary rehabilitation
RR: Respiratory rate
SD: Standard deviation
SEM: Sensor electronics module
SpO_2_: Oxygen saturation
ST: Skin temperature
WT: Wear time
