# Supplementary material for: A proof of concept for continuous, non-invasive, free-living vital signs monitoring to predict readmission following an acute exacerbation of COPD: a prospective cohort study
Source: Respir Res. 2022 Apr 26;23:102. doi: 10.1186/s12931-022-02018-5 (PMC9044843; doi:10.1186/s12931-022-02018-5)
Supplement: Supplementary file 1 — Additional file 1: Figure S2. Individual plots for respiratory rate, heart rate, skin temperature and physical activity for all participants. [file 12931_2022_2018_MOESM1_ESM.docx]

**Supplementary Figure S2:** Individual plots for RR, HR, Skin Temp and PA for all participants. Patients 1-14 experienced an AECOPD during the 6-week study period, and patients 15-31 had no AECOPD.

**Abbreviations:** EXACT: Exacerbations of Chronic Pulmonary Disease Tool; HR: Heart rate; PA: Physical activity; RR: Respiratory rate; Skin Temp: Skin temperature.

**
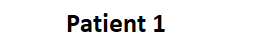
**
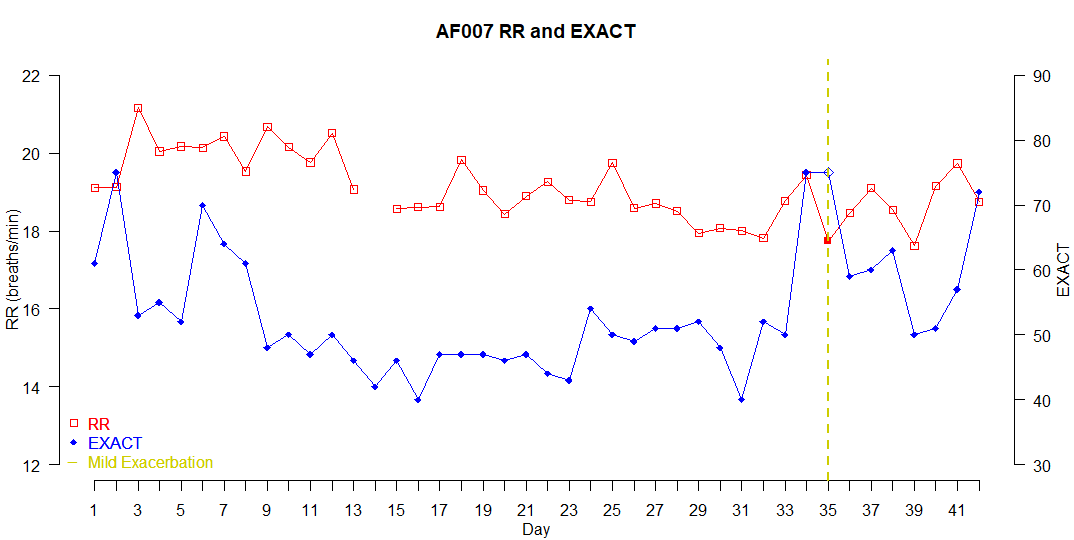
**AECOPD group**


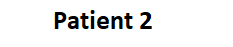

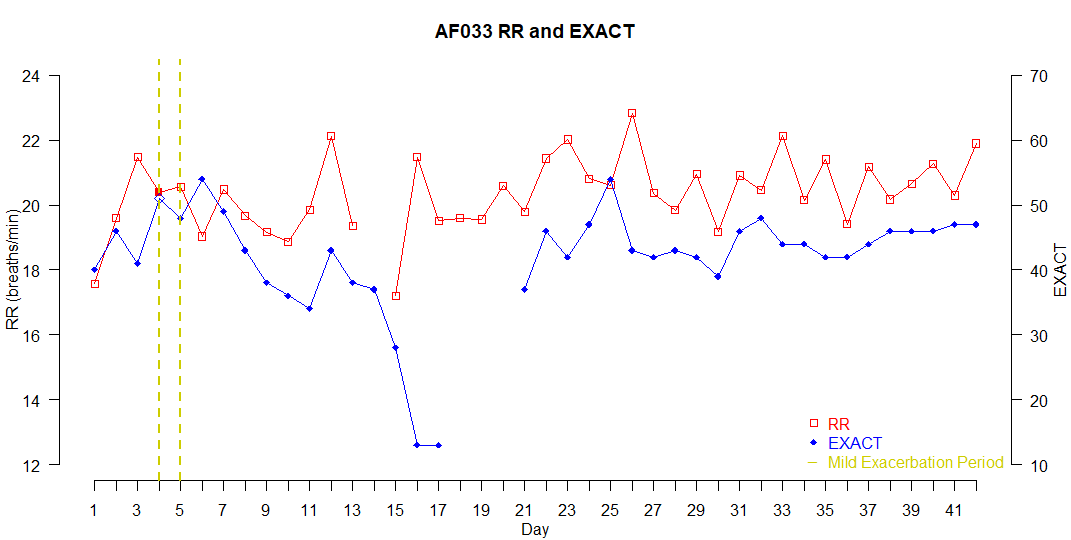


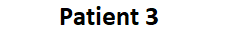

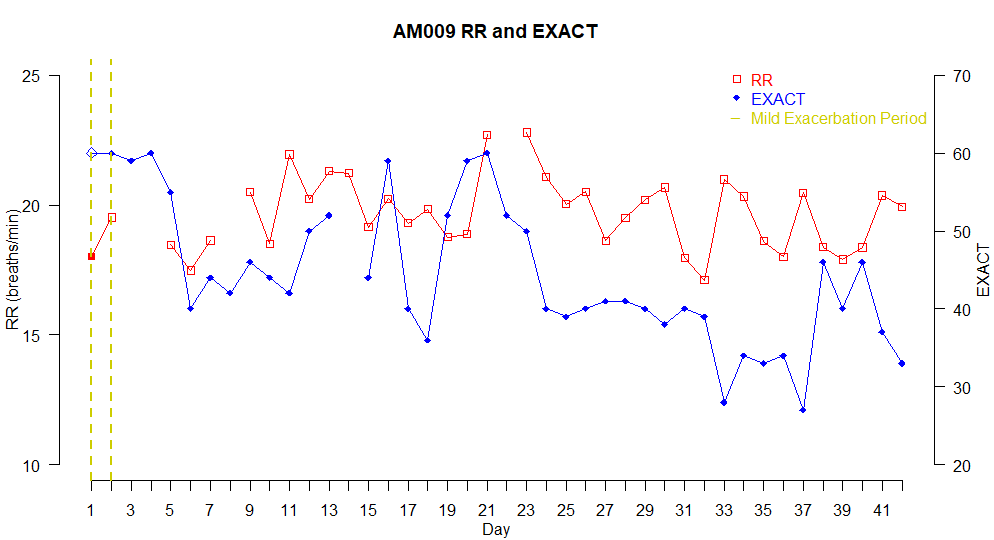


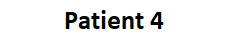

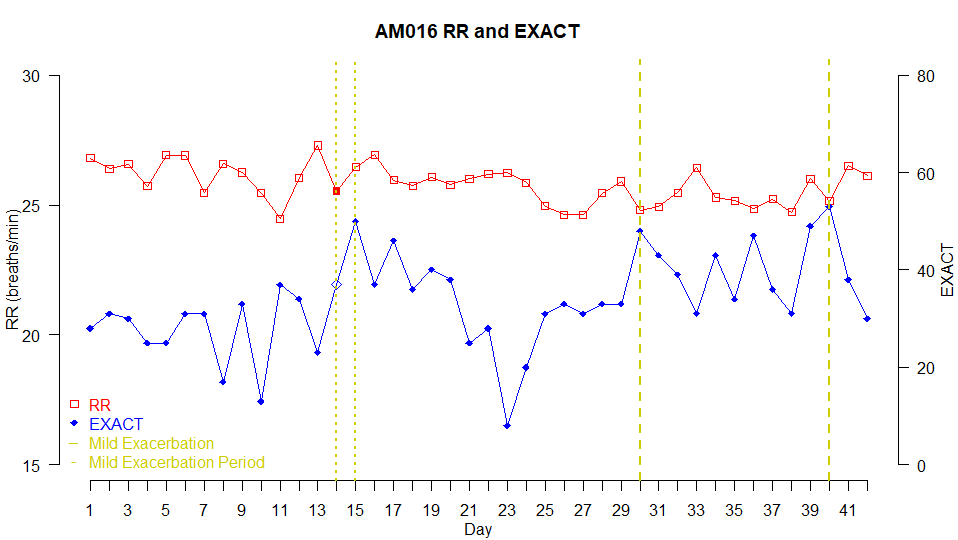


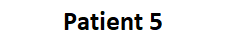

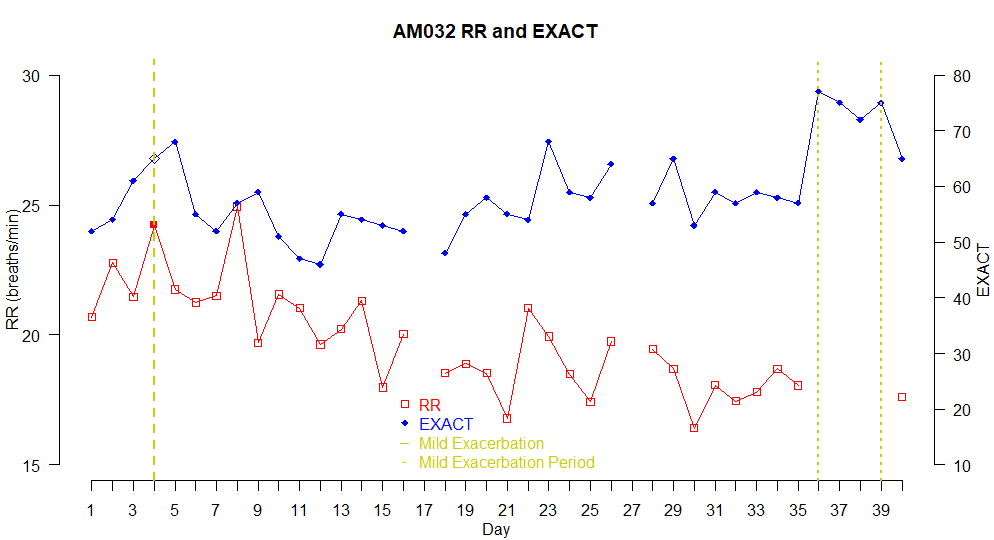


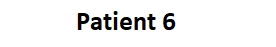

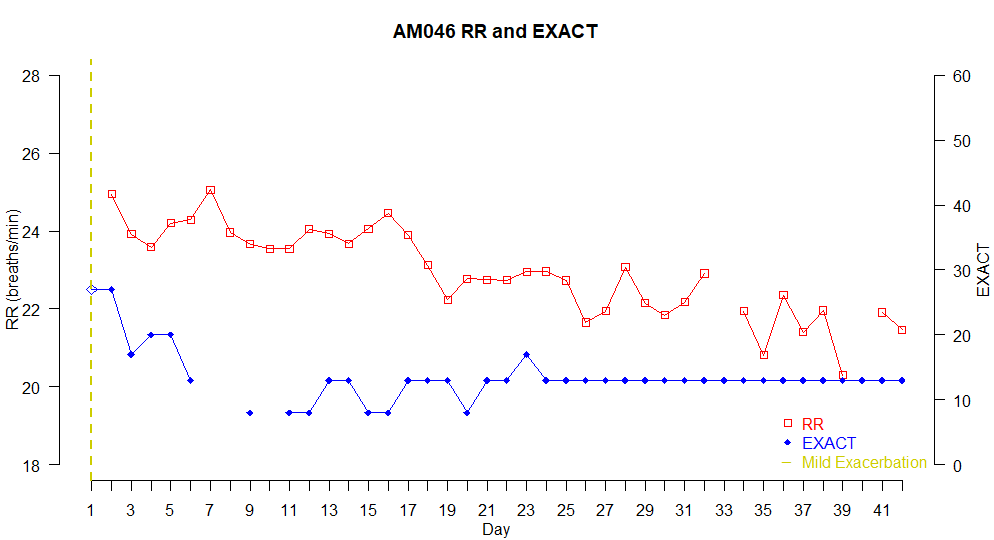


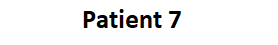

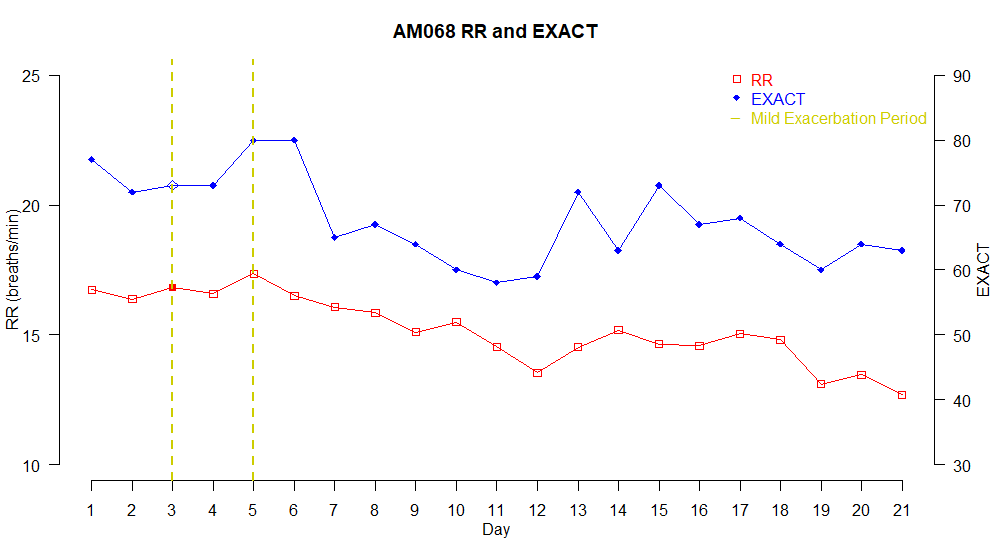


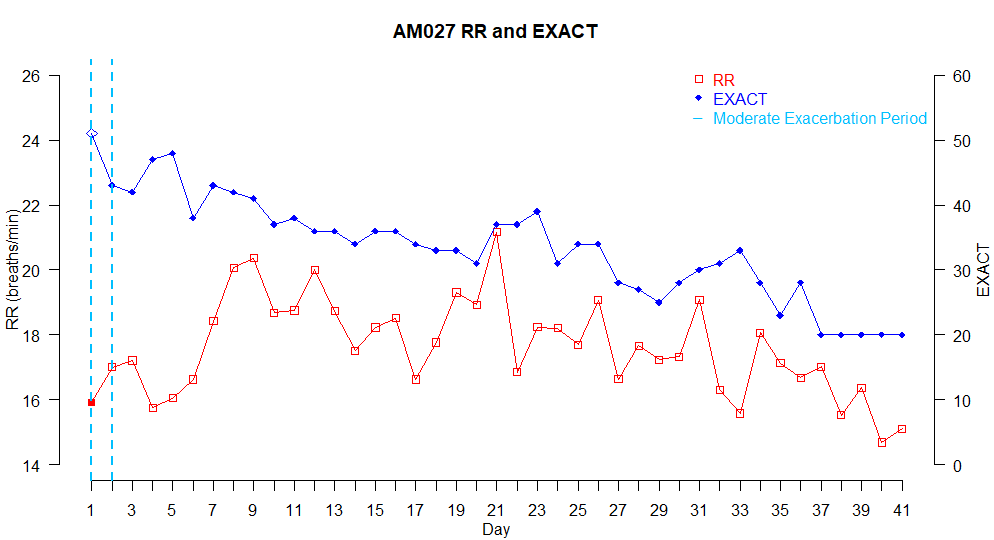


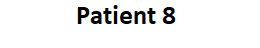


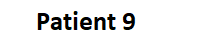


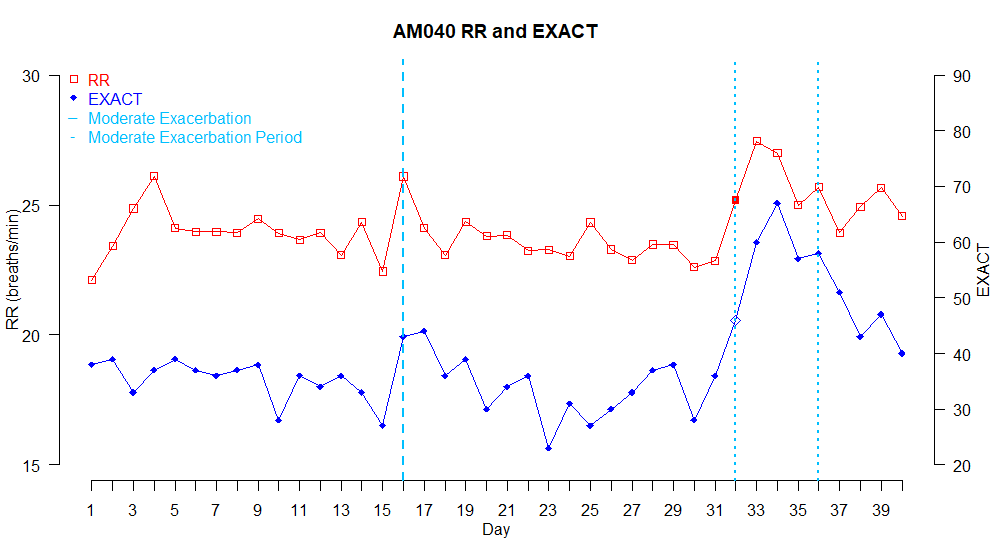


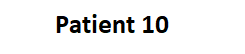

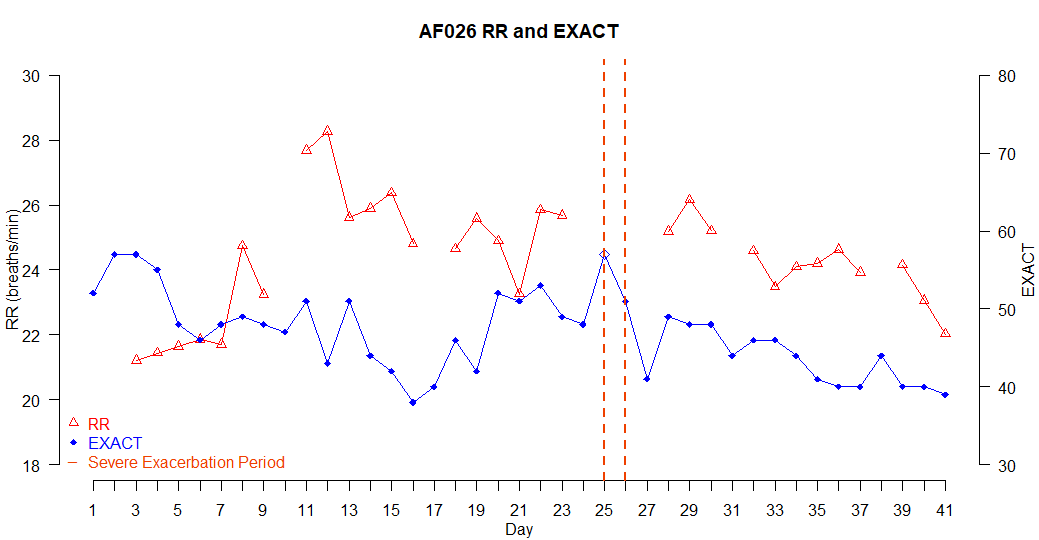


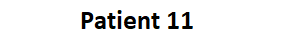

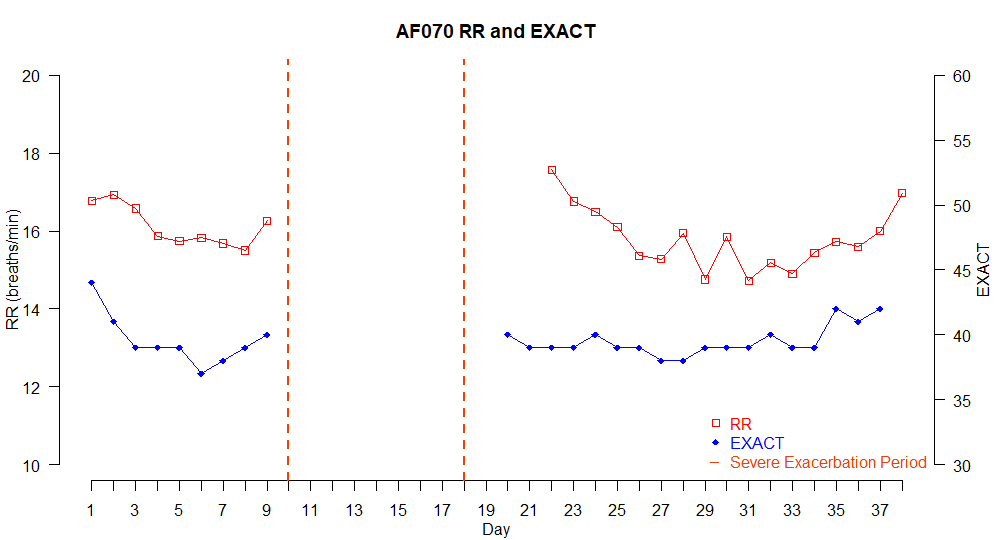


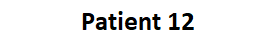

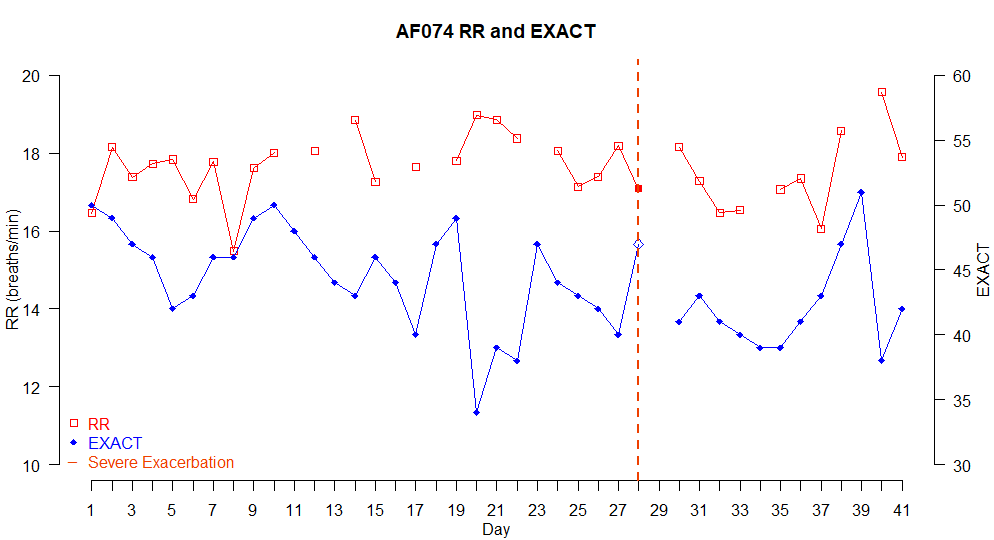


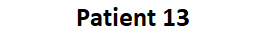

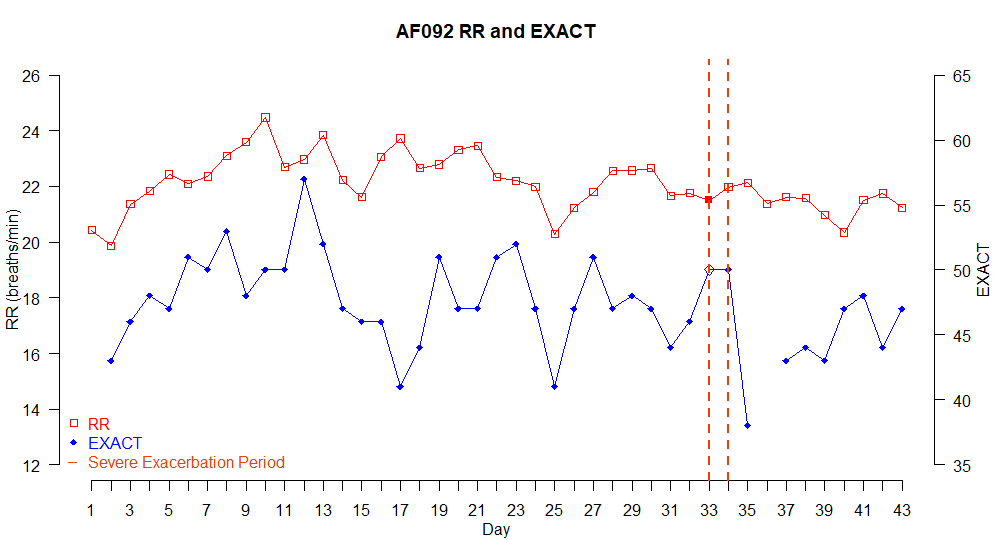


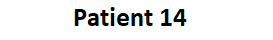

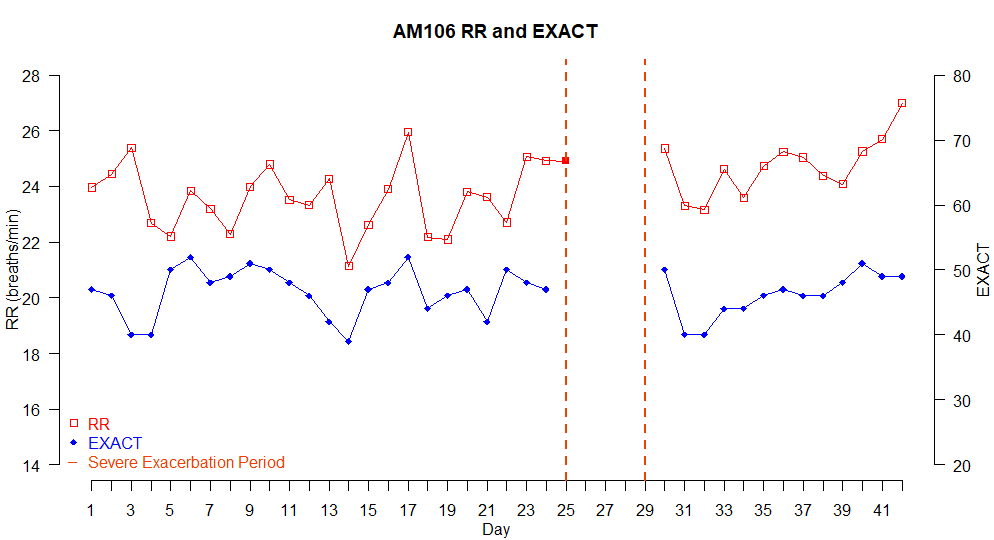


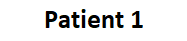


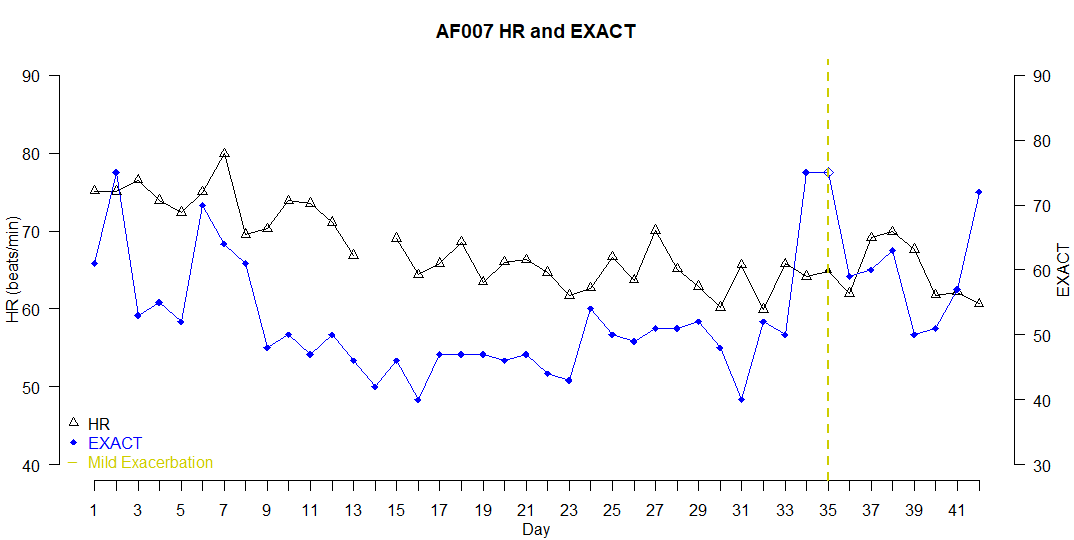


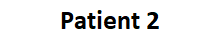

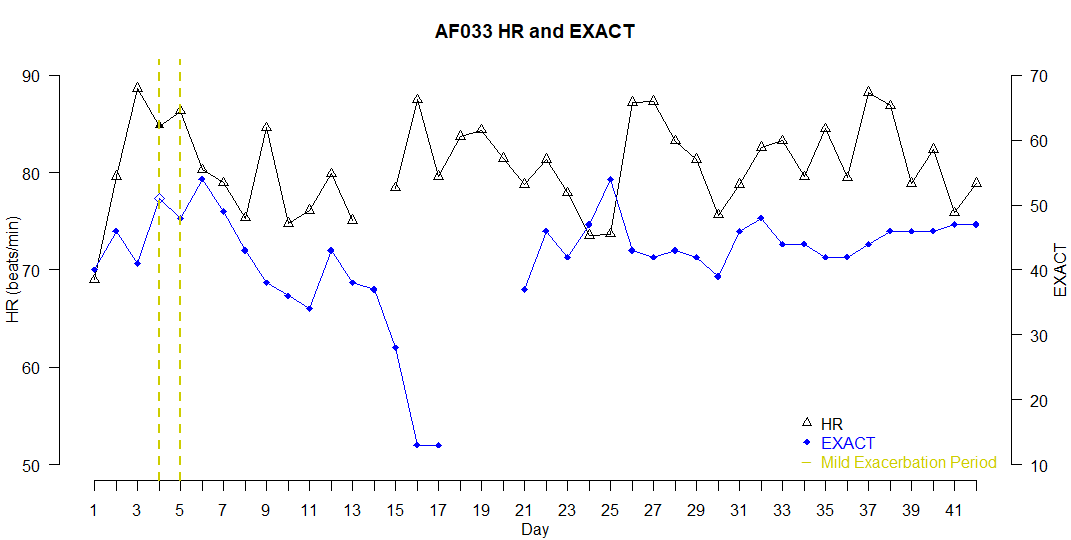


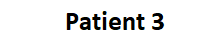

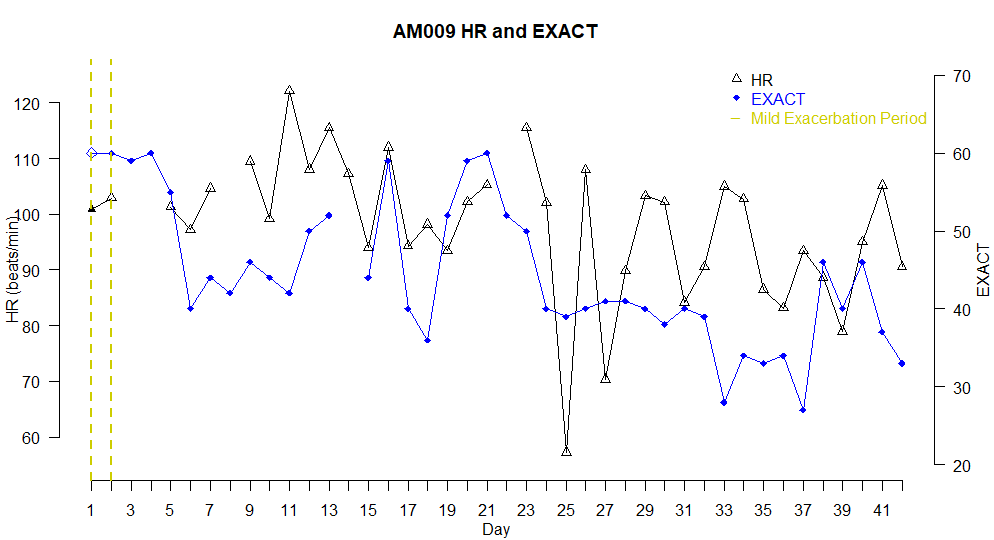


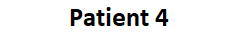

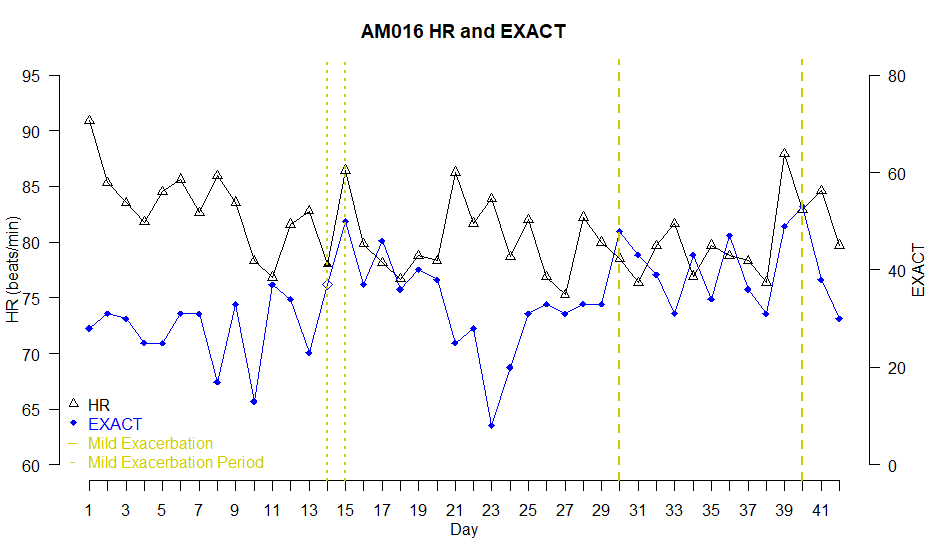


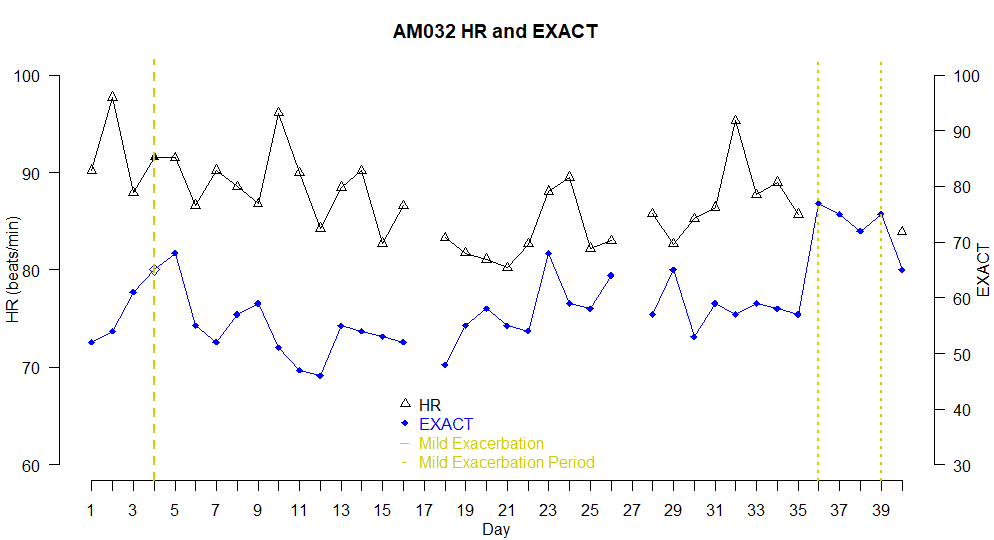


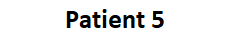

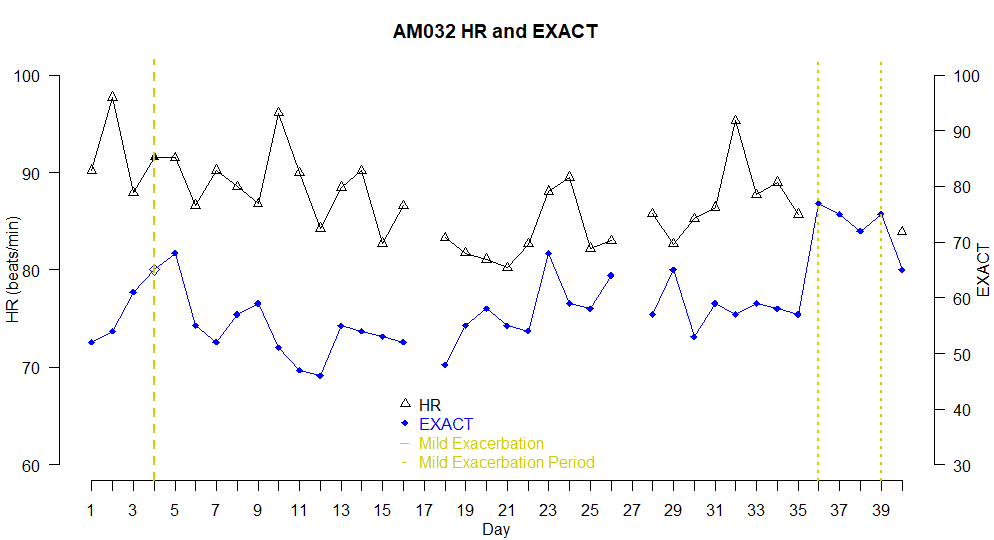


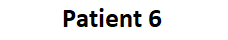

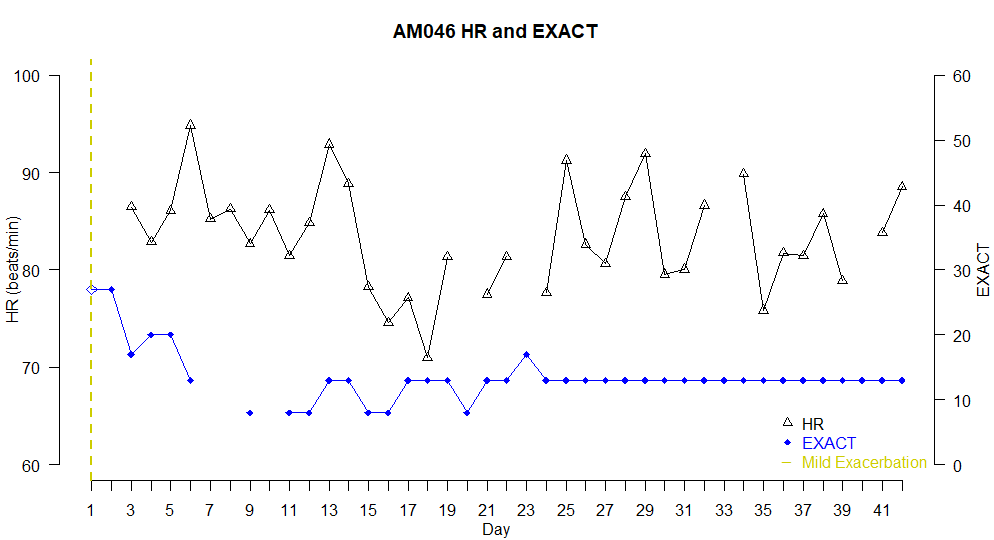


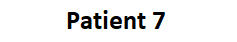

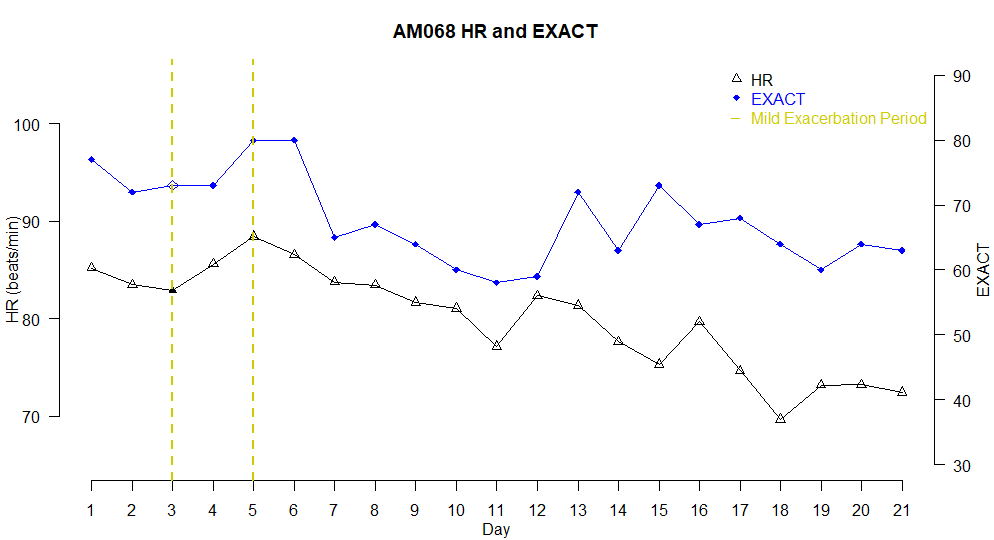


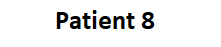

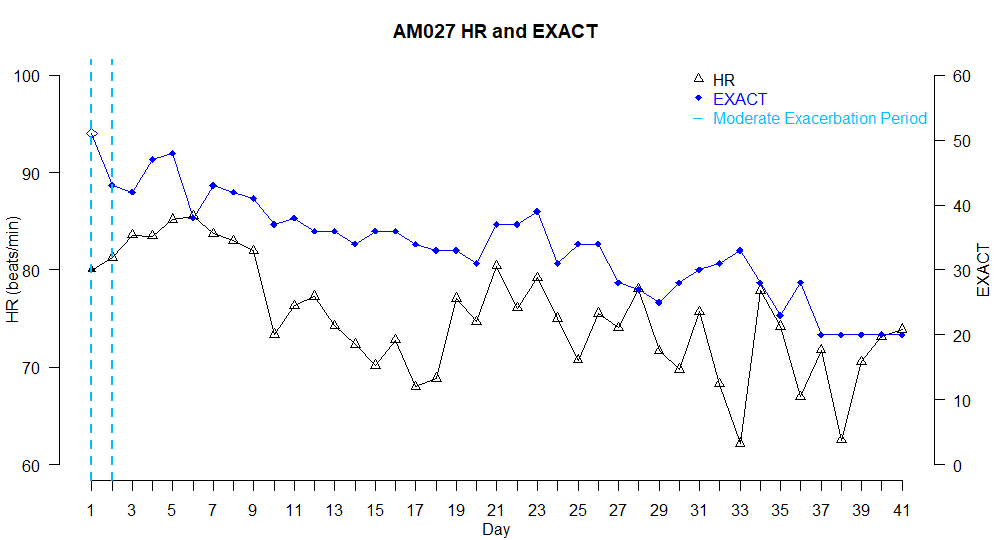


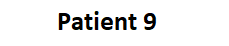

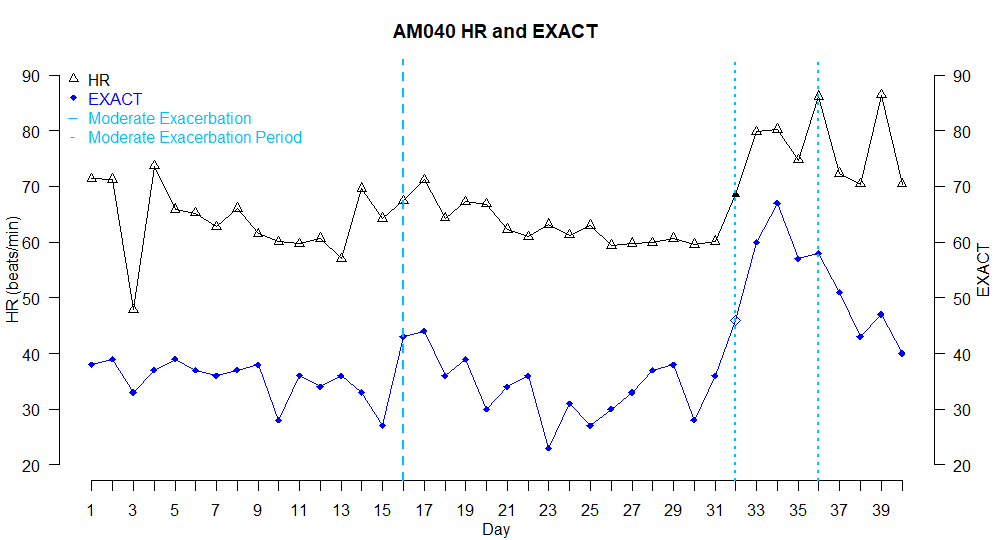


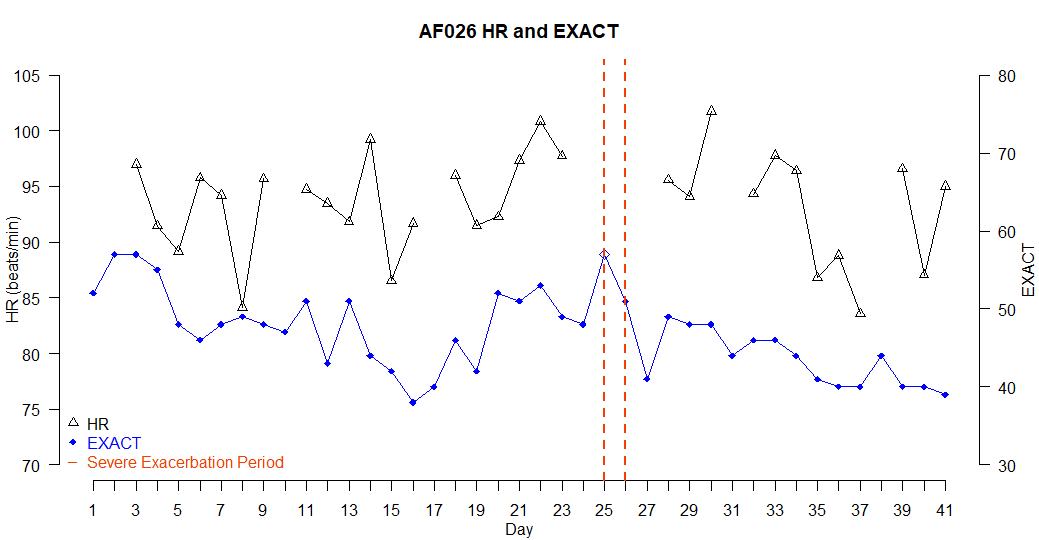


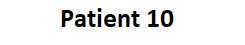


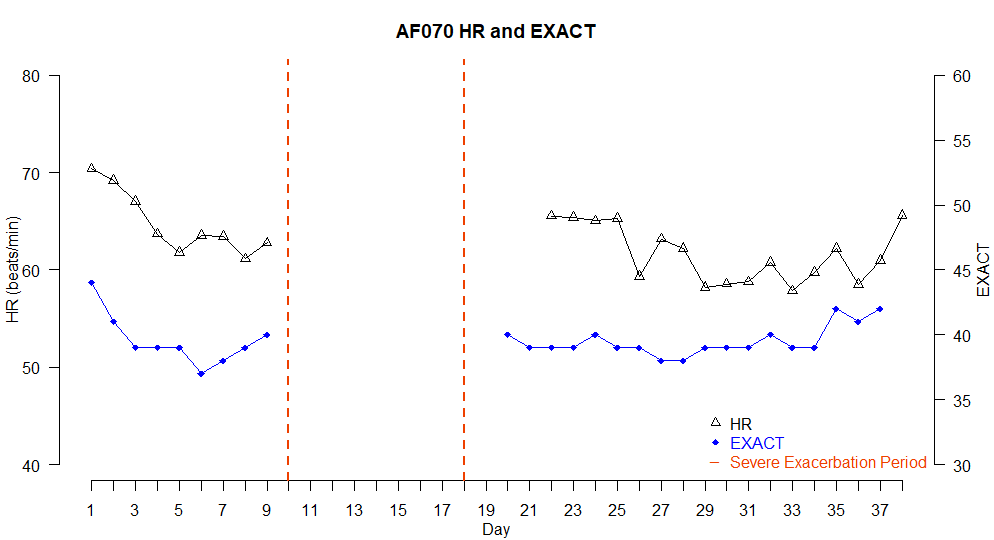


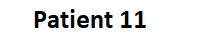


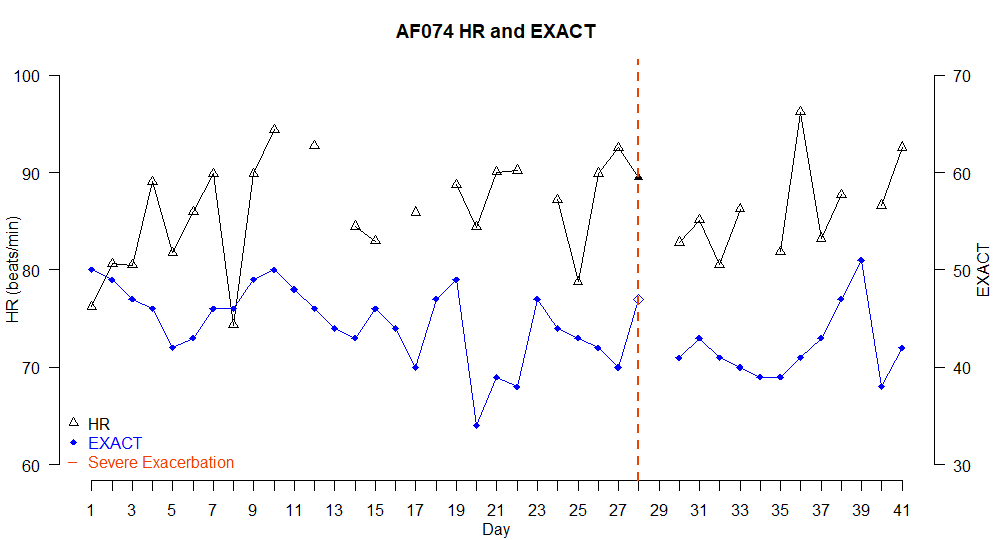

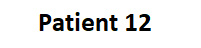


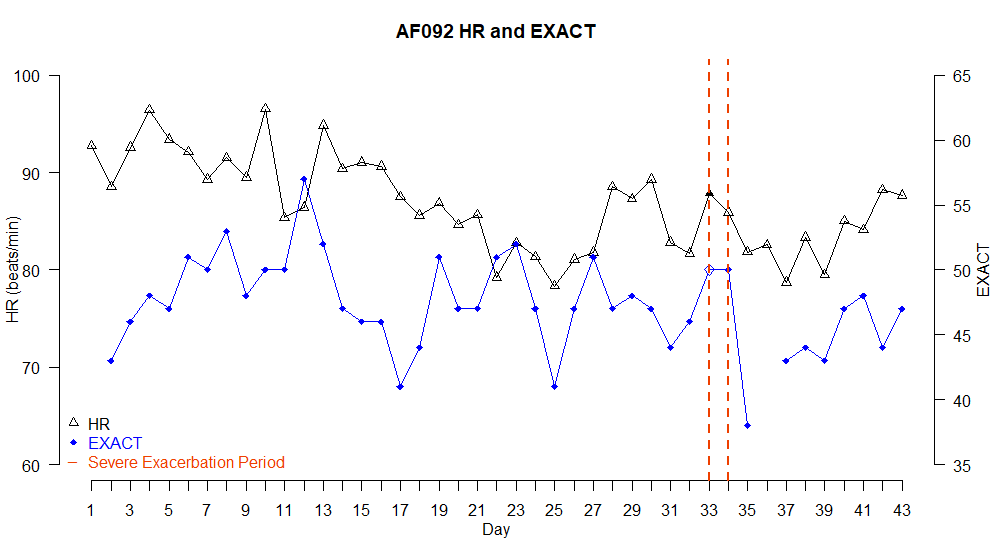


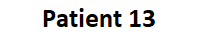


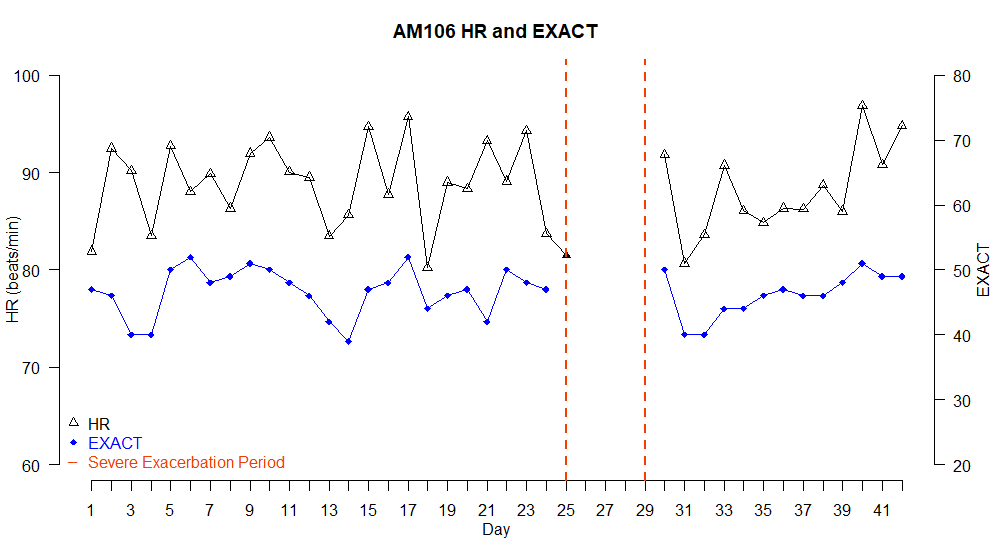

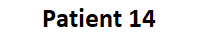


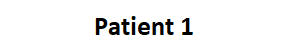

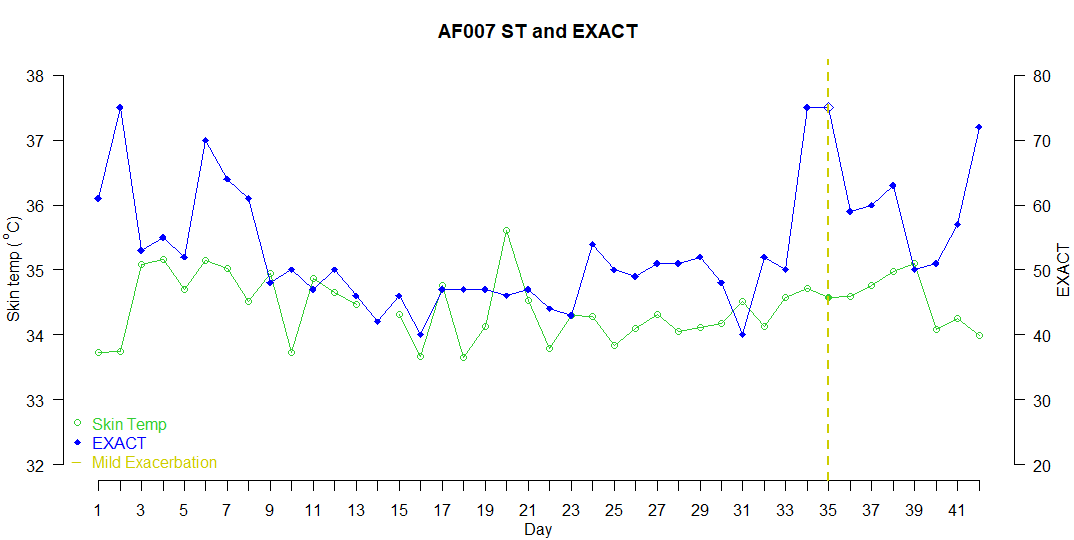


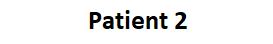

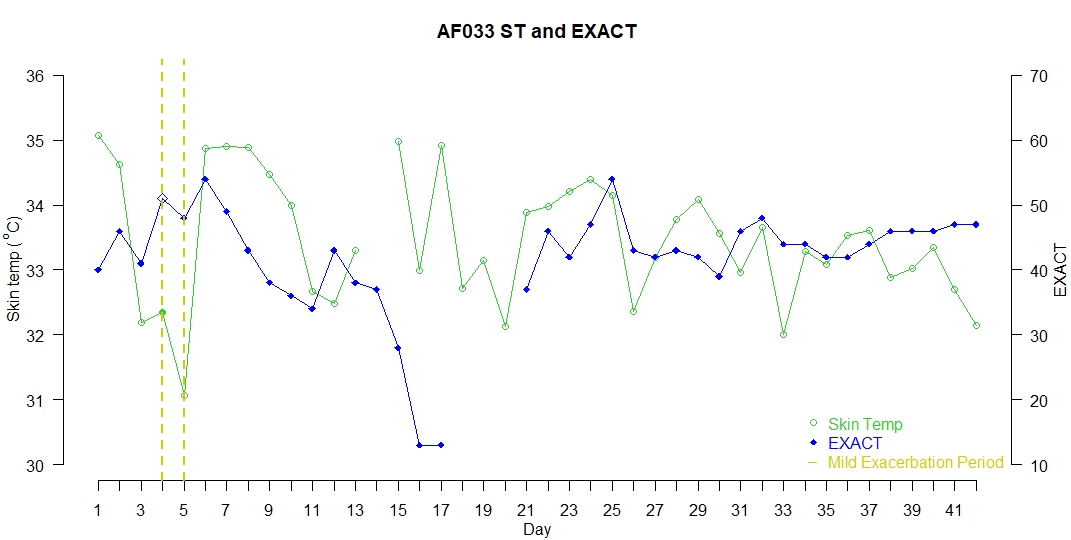


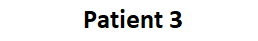

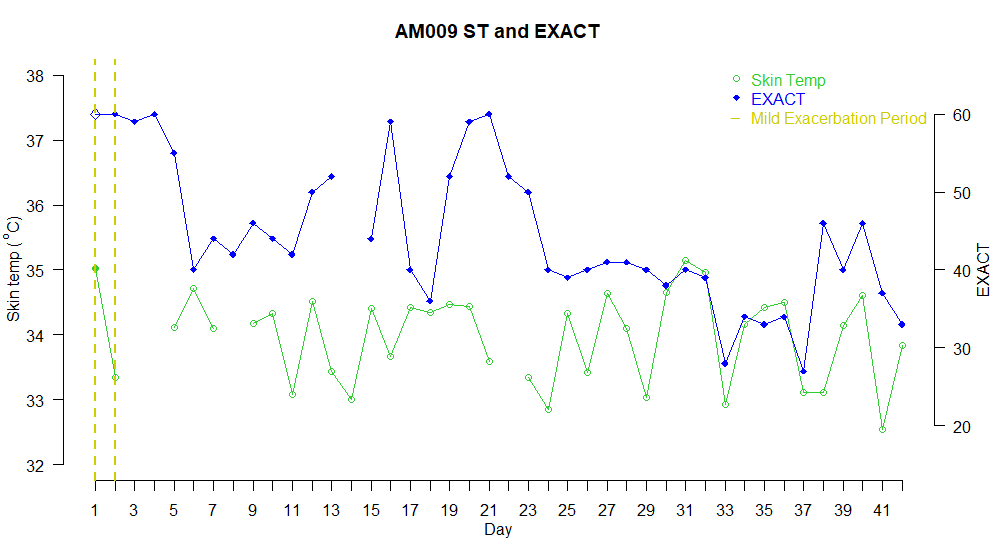


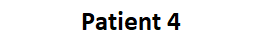


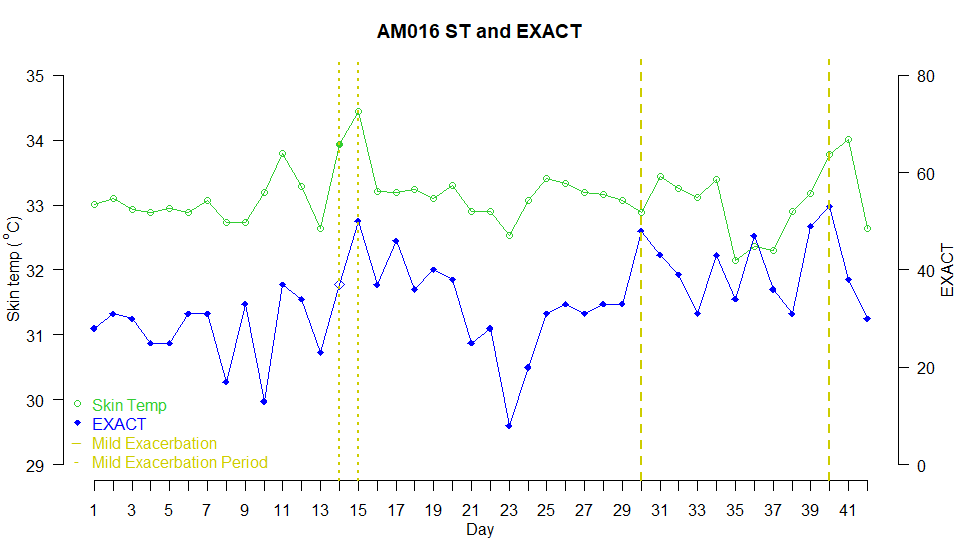


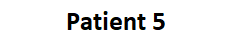


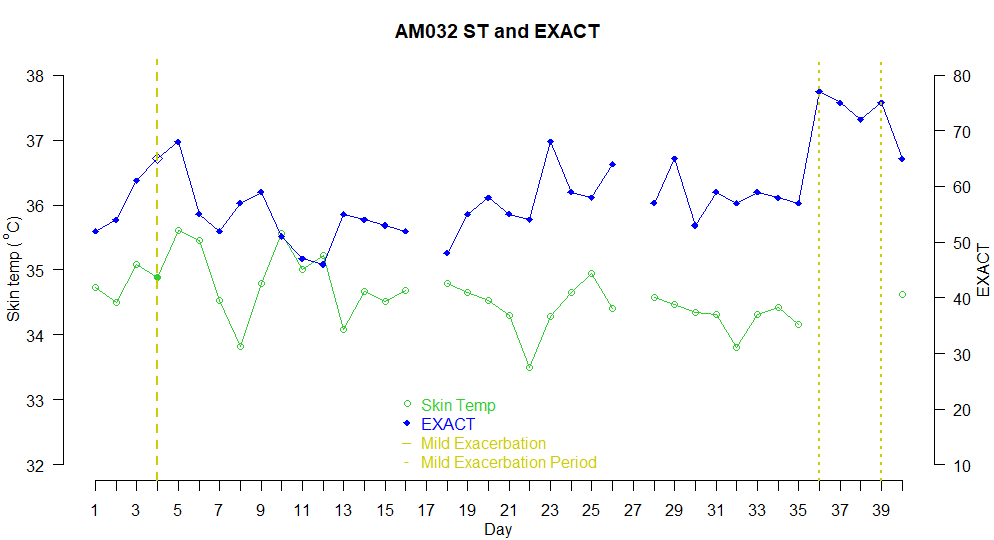


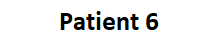


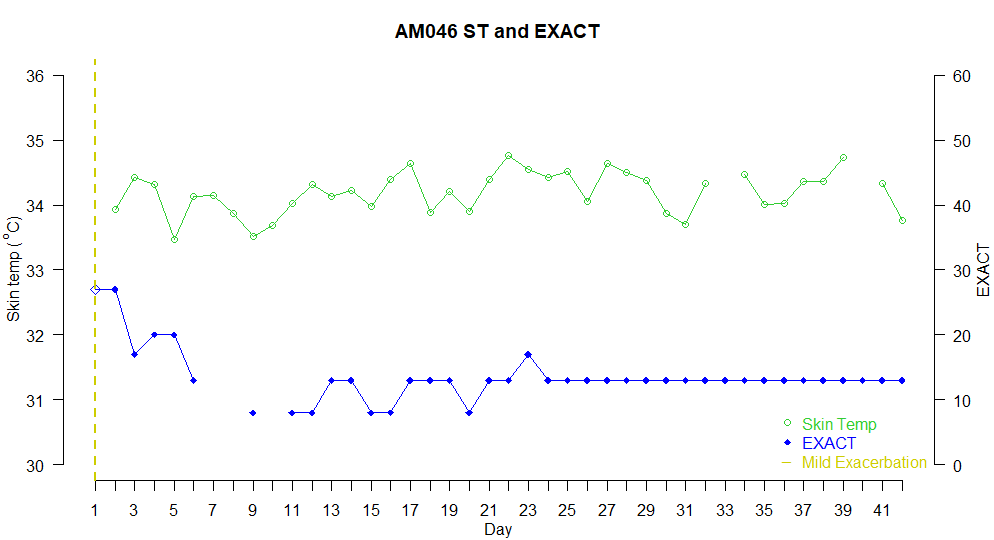


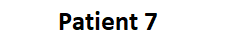


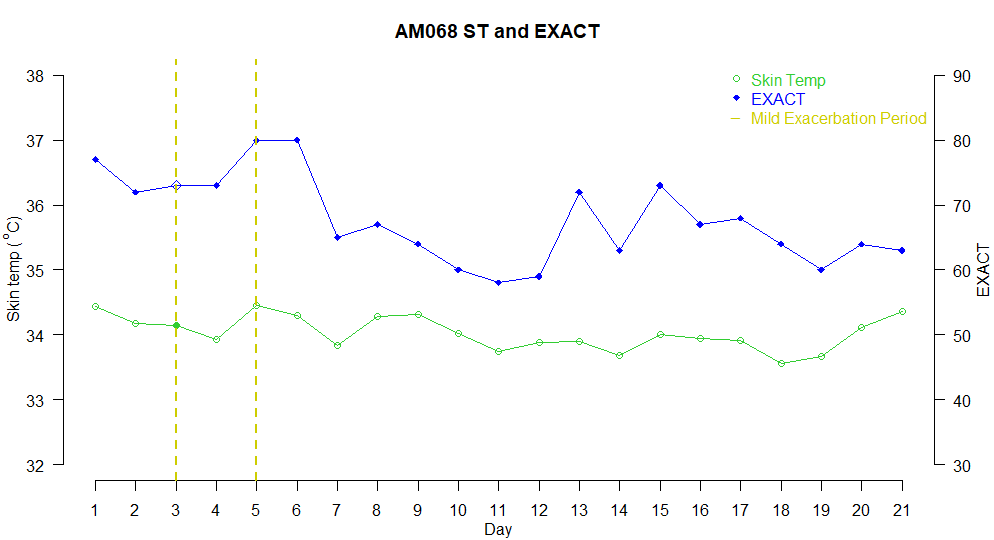


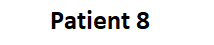

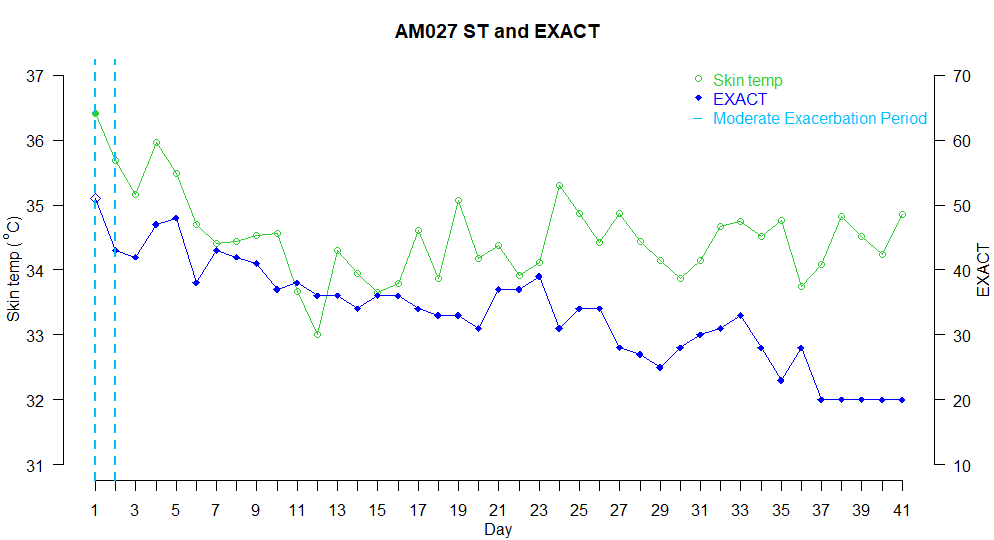


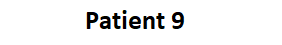

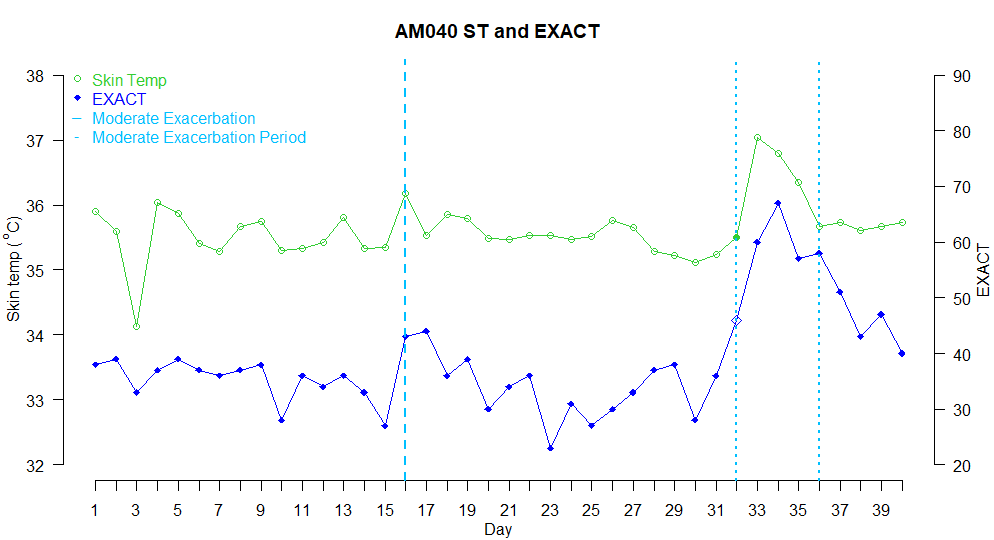


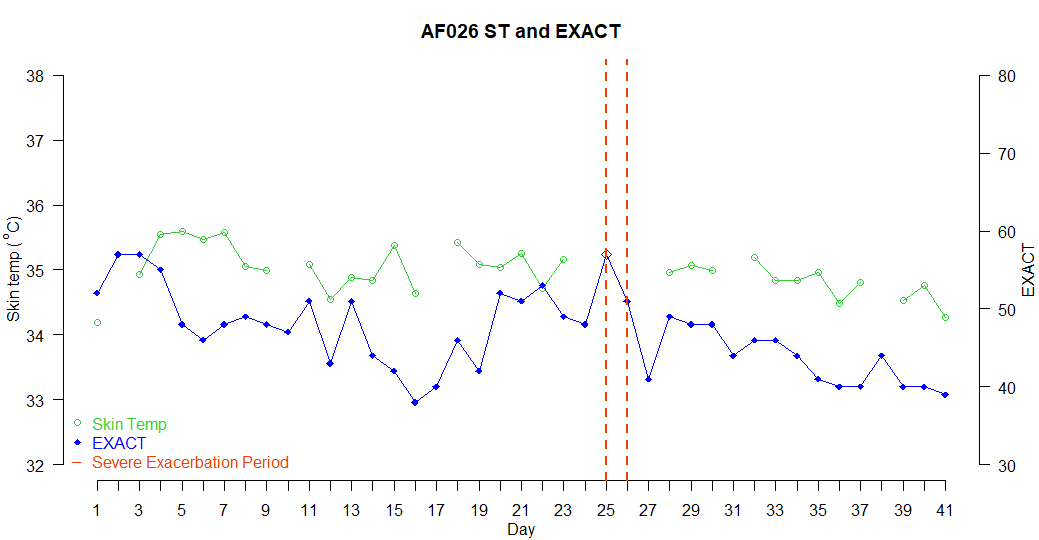


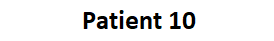


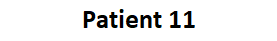

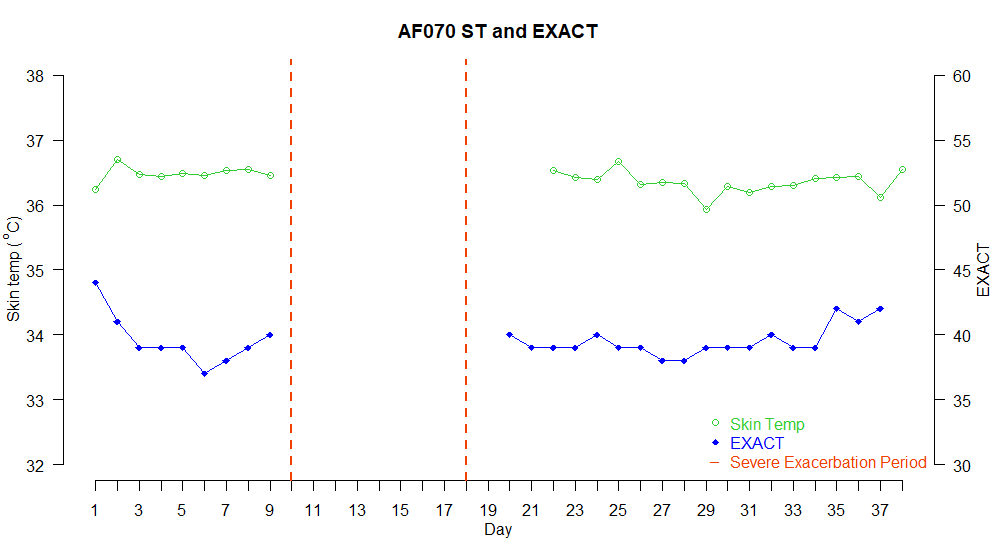


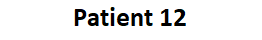

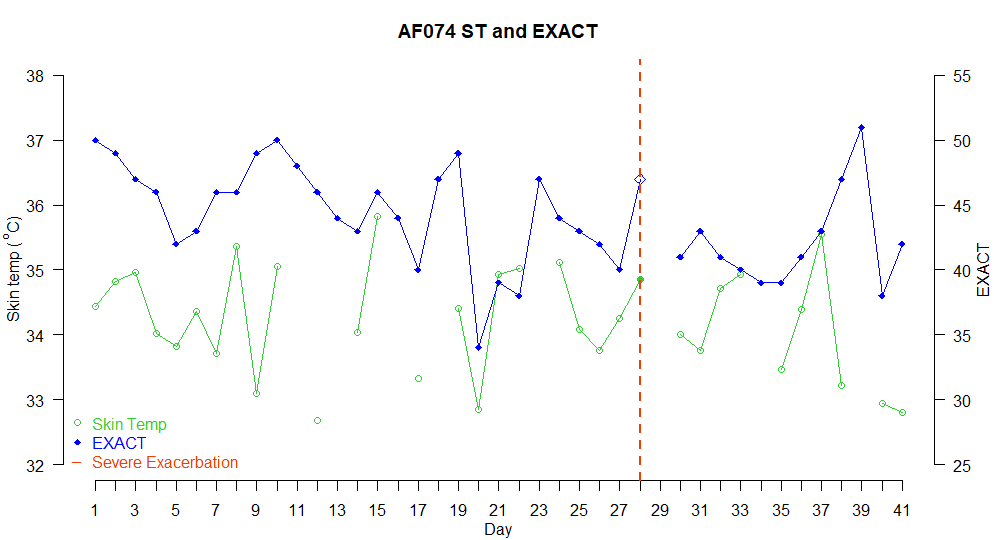


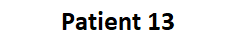

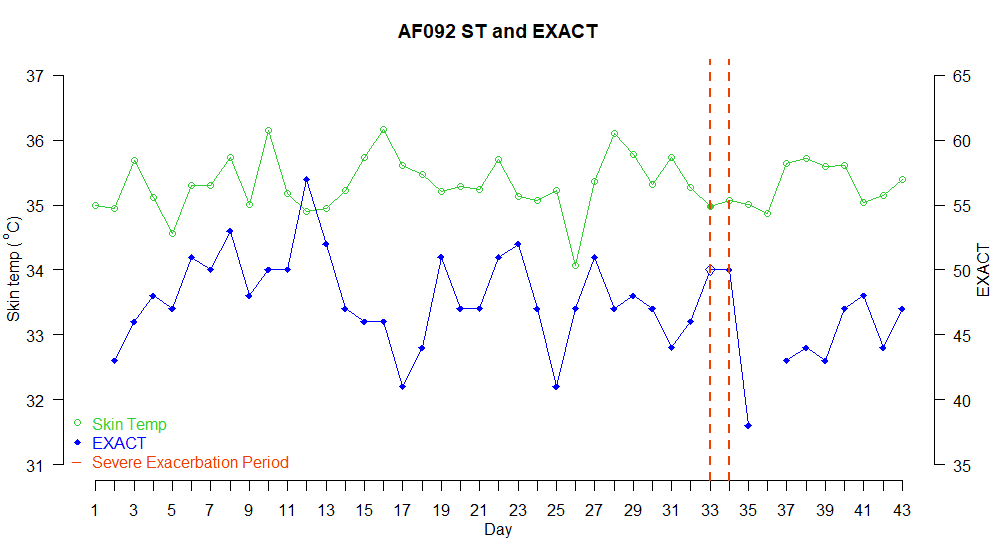


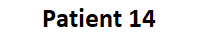

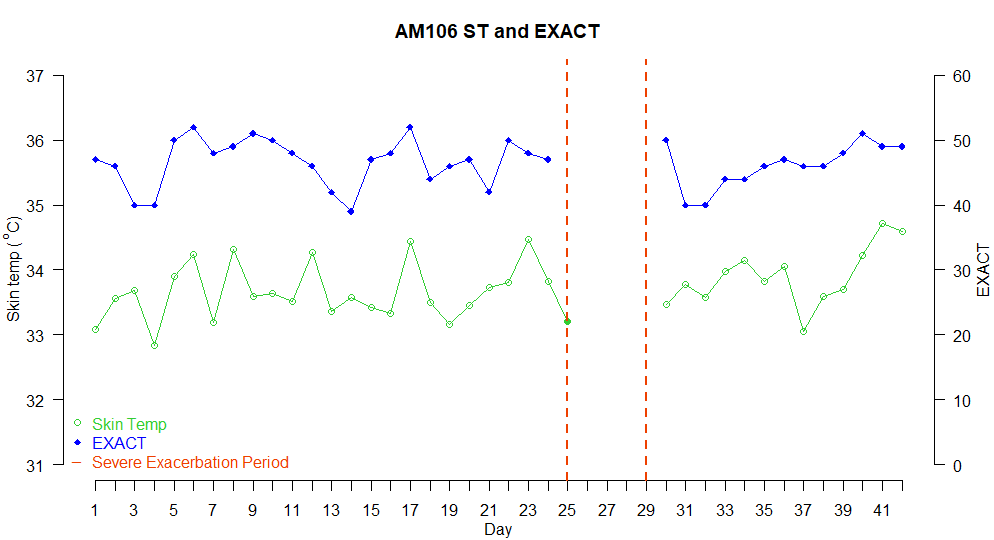


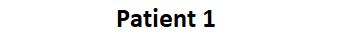


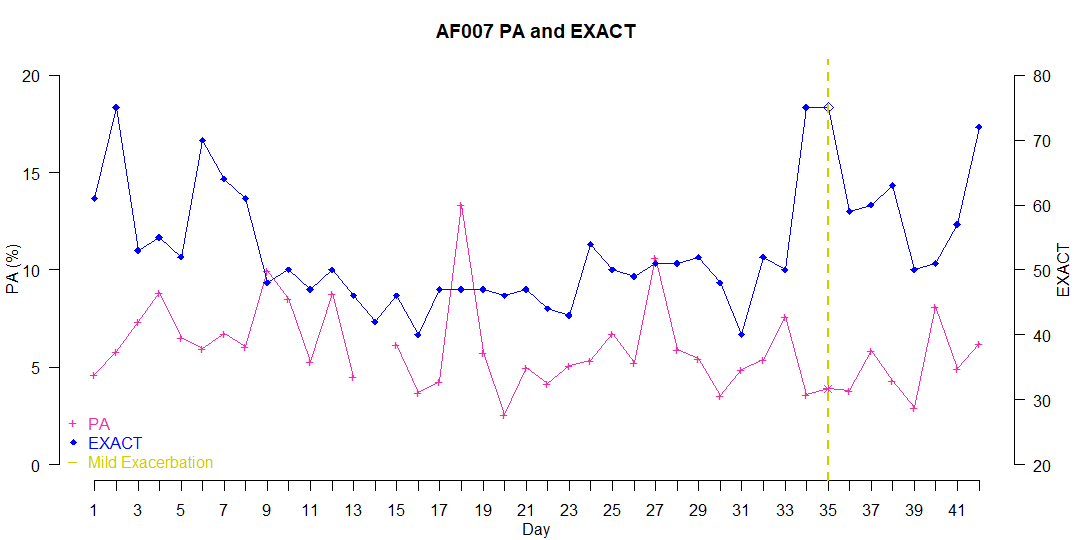


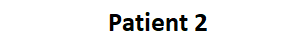

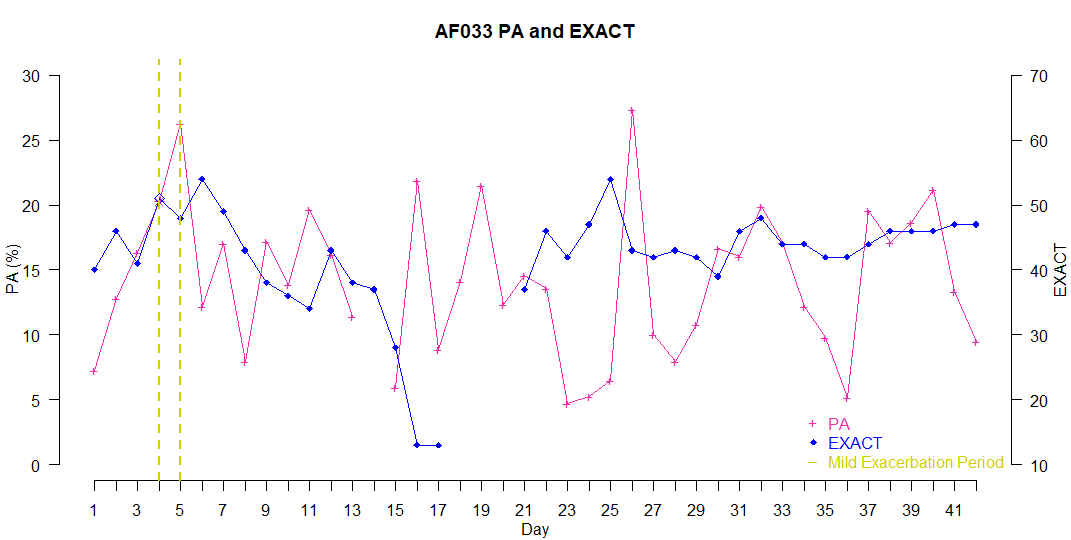


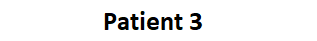

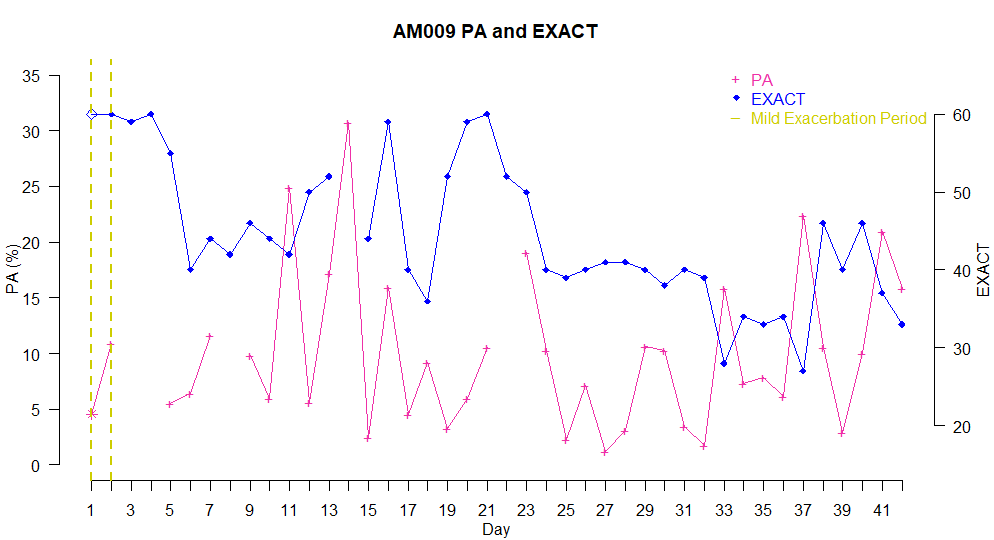


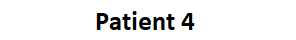


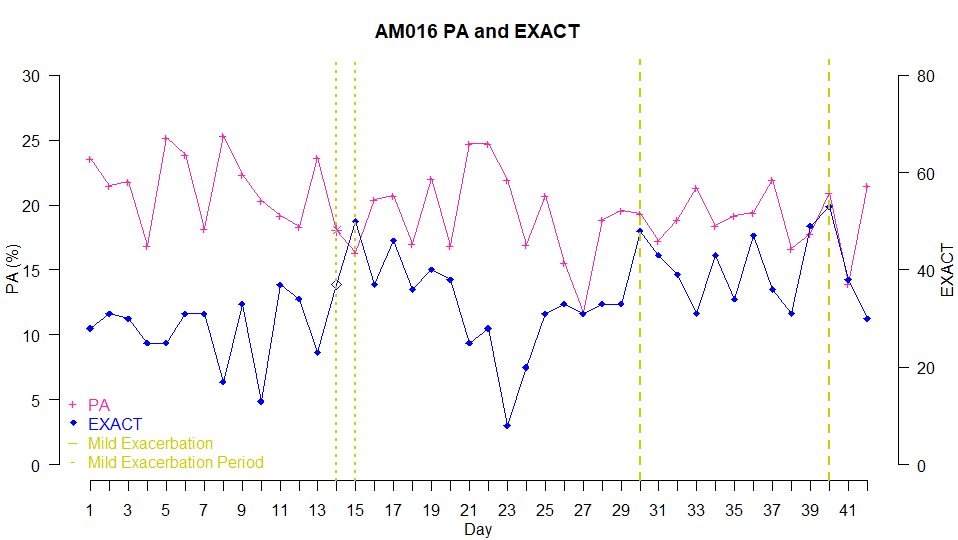


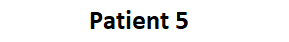

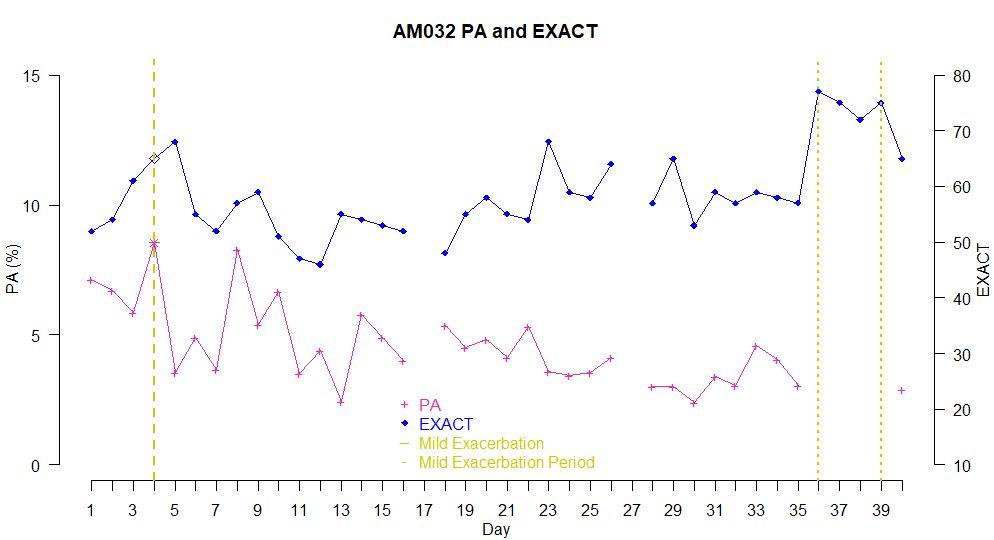


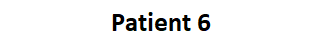

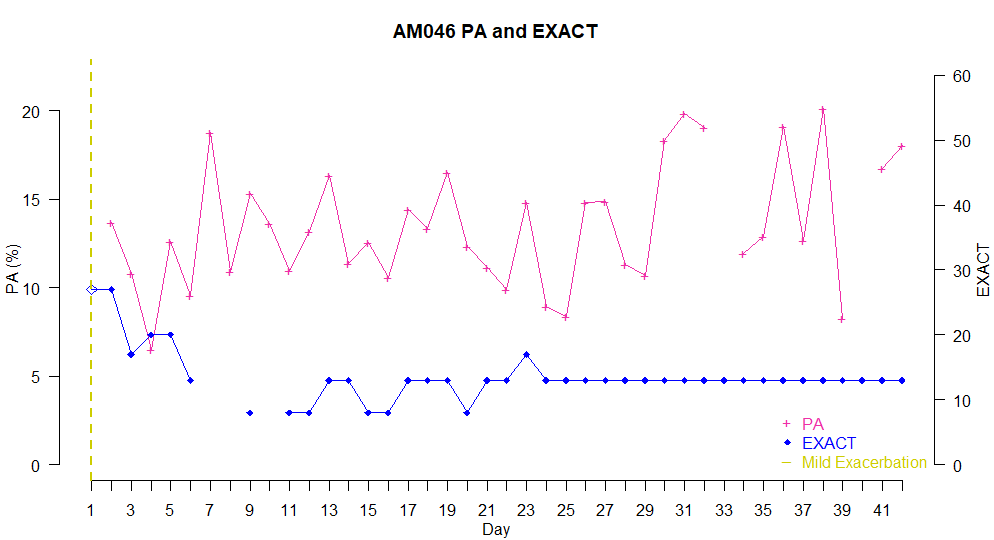


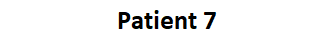

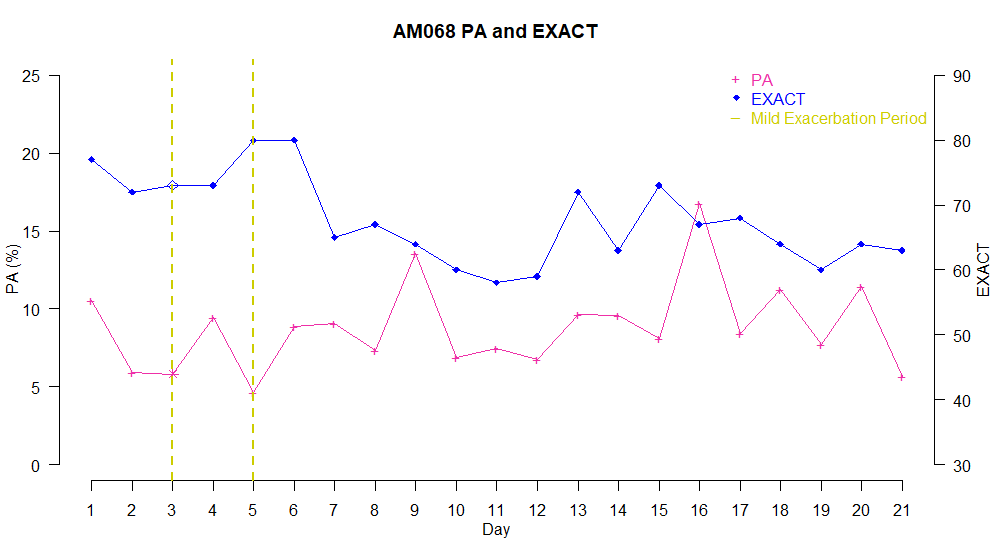


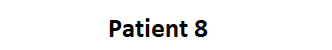


**No AECOPD group**
