# Supplementary material for: A proof of concept for continuous, non-invasive, free-living vital signs monitoring to predict readmission following an acute exacerbation of COPD: a prospective cohort study
Source: Respir Res. 2022 Apr 26;23:102. doi: 10.1186/s12931-022-02018-5 (PMC9044843; doi:10.1186/s12931-022-02018-5)
Supplement: Supplementary file 2 — Additional file 2: Table S1. Linear Mixed Model for all variables and EXACT score. [file 12931_2022_2018_MOESM2_ESM.docx]

**Supplementary Table S1:** Linear Mixed Model for all variables and EXACT score

Linear mixed model fit by REML. t-tests use Satterthwaite's method ['lmerModLmerTest']

Formula: EXACT_Score ~ BR + HR + ST + PA + Time_Point + (1 | Pat_ID)

Data: dd_all

REML criterion at convergence: 5788.8

Scaled residuals:

Min 1Q Median 3Q Max

-6.1982 -0.4974 0.0324 0.5250 3.4060

Random effects:

Groups Name Variance Std.Dev.

Pat_ID (Intercept) 136.86 11.699

Residual 50.04 7.074

Number of obs: 839, groups: Pat_ID, 28

Fixed effects:

Estimate Std. Error df t value Pr(>|t|)

(Intercept) -9.84504 17.84747 828.81854 -0.552 0.581357

BR 0.25255 0.19911 775.78133 1.268 0.205043

HR 0.26662 0.04761 832.96218 5.601 2.9e-08 ***

ST 0.83445 0.50636 831.66278 1.648 0.099741 .

PA -0.22383 0.06675 823.84454 -3.353 0.000835 ***

Time_Point -0.08490 0.02245 822.70918 -3.781 0.000167 ***

---

Signif. codes: 0 ‘***’ 0.001 ‘**’ 0.01 ‘*’ 0.05 ‘.’ 0.1 ‘ ’ 1

Correlation of Fixed Effects:

(Intr) BR HR ST PA

BR -0.146

HR -0.015 -0.276

ST -0.954 -0.028 -0.140

PA -0.357 -0.189 -0.222 0.422

Time_Point -0.126 0.276 0.069 0.030 -0.141

2.5 % 97.5 %

.sig01 8.9099406 15.35606205

.sigma 6.7229318 7.41065025

(Intercept) -44.6630597 25.20163485

BR -0.1430625 0.64207244

HR 0.1737585 0.36032828

ST -0.1583117 1.82295698

PA -0.3549337 -0.09357597

Time_Point -0.1290018 -0.04108353
