# Supplementary material for: A proof of concept for continuous, non-invasive, free-living vital signs monitoring to predict readmission following an acute exacerbation of COPD: a prospective cohort study
Source: Respir Res. 2022 Apr 26;23:102. doi: 10.1186/s12931-022-02018-5 (PMC9044843; doi:10.1186/s12931-022-02018-5)
Supplement: Supplementary file 3 — Additional file 3: Table S3. Collective summary of individual participant data, 3 days-, 2 days- and the day before the onset of exacerbation. [file 12931_2022_2018_MOESM3_ESM.docx]

**Supplementary Table S3:** Collective summary of individual participant data, 3 days-, 2 days- and the day before the onset of exacerbation

|  | **RR (breaths/min)** | | | | **HR (bpm)** | | | | **ST (°C)** | | | | **PA (min)** | | | |
| --- | --- | --- | --- | --- | --- | --- | --- | --- | --- | --- | --- | --- | --- | --- | --- | --- |
| **Participant ID** | **E-3 to E** | **E-2 to E** | **E-1 to E** | **E_S_ - E_F_** | **E-3 to E** | **E-2 to E** | **E-1 to E** | **E_S_ - E_F_** | **E-3 to E** | **E-2 to E** | **E-1 to E** | **E_S_ - E_F_** | **E-3 to E** | **E-2 to E** | **E-1 to E** | **E_S_ - E_F_** |
| 1 | -0.1 | -1.2 | -1.8 | NA | 6.7 | -1.2 | 0.8 | NA | 0.45 | -0.01 | -0.14 | NA | -20.7 | -36.5 | 6.5 | NA |
| 2 | 3.3 | 0.8 | -1.1 | 0.2 | 18.8 | 5.3 | -3.6 | 1.5 | -2.69 | -2.28 | 0.14 | -1.35 | 134.4 | 43.5 | 17.8 | 22.2 |
| 3 | NA | NA | NA | 1.7 | NA | NA | NA | 1.6 | NA | NA | NA | -1.66 | NA | NA | NA | 104.4 |
| 4a | 0.9 | -0.4 | -1.3 | 0.8 | 1.2 | -3.7 | -4.8 | 9.0 | 0.14 | 0.69 | 1.38 | 0.52 | -4.4 | -1.4 | -17.9 | -7.1 |
| 4b | 0.1 | -0.5 | -0.9 | NA | 3.6 | -3.7 | -1.5 | NA | -0.31 | -0.28 | -0.17 | NA | 49.0 | 1.9 | -0.7 | NA |
| 4c | -0.1 | 0.3 | -0.7 | NA | 4.8 | 6.5 | -4.7 | NA | 1.59 | 0.93 | 0.62 | NA | -3.4 | 17.2 | 13.4 | NA |
| 5a | 3.5 | 1.3 | 2.6 | NA | 1.2 | -5.2 | 3.4 | NA | 0.14 | 0.38 | -0.21 | NA | 14.7 | 20.7 | 34.1 | NA |
| 5b | M | M | M | M | M | M | M | M | M | M | M | M | M | M | M | M |
| 6 | NA | NA | NA | NA | NA | NA | NA | NA | NA | NA | NA | NA | NA | NA | NA | NA |
| 7 | NA | 0.1 | 0.6 | 0.7 | NA | -2.2 | -0.6 | 5.5 | NA | -0.31 | -0.03 | 0.31 | NA | -33.6 | -1.1 | -14.9 |
| 8 | NA | NA | NA | 1.4 | NA | NA | NA | 1.3 | NA | NA | NA | -0.69 | NA | NA | NA | -19.0 |
| 9 | 2.7 | 1.5 | 3.4 | NA | 15.0 | -2.6 | 4.0 | NA | 0.35 | 0.83 | 0.83 | NA | 38.3 | -24.7 | -2.5 | NA |
| 9b | 1.5 | 2.4 | 2.1 | 0.4 | 10.6 | 12.3 | 11.4 | 21.1 | 0.28 | 0.38 | 0.28 | 0.14 | 34.7 | 37.5 | 7.7 | -8.4 |
| 10 | M | M | M | M | M | M | M | M | M | M | M | M | M | M | M | M |
| 11 | M | M | M | M | M | M | M | M | M | M | M | M | M | M | M | M |
| 12 | -0.1 | -0.4 | -1.3 | NA | 11.1 | -0.4 | -2.7 | NA | 0.76 | 1.10 | 0.59 | NA | -20.4 | -43.6 | -42.6 | NA |
| 13 | -1.0 | -0.1 | -0.2 | 0.5 | -1.3 | 4.9 | 6.2 | -1.8 | -0.31 | -0.72 | -0.28 | 0.07 | -22.4 | 132.2 | 81.9 | 27.5 |
| 14 | 2.0 | -0.1 | 0.0 | M | -7.1 | -11.2 | -2.2 | M | -0.62 | -1.28 | -0.62 | M | -37.5 | -46.9 | -1.7 | M |

**Notes:** Mild, moderate, and severe exacerbations are represented as, yellow, blue and red, respectively.

**Abbreviations:** E=exacerbation; E_F_= End of exacerbation; E_S_=onset of exacerbation; HR: Heart rate; M=Missing data (Equivital LifeMonitor not worn); NA=not available; PA: Physical activity; RR: Respiratory rate; ST: Skin temperature.
